# Risk factors for overweight and obesity after childhood acute lymphoblastic leukemia in North America and Switzerland: A comparison of two cohort studies

**DOI:** 10.1002/cam4.6588

**Published:** 2023-10-09

**Authors:** Fabiën N. Belle, Christina Schindera, Marc Ansari, Gregory T. Armstrong, Maja Beck‐Popovic, Rebecca Howell, Wendy M. Leisenring, Lillian R. Meacham, Jochen Rössler, Ben D. Spycher, Emily Tonorezos, Nicolas X. von der Weid, Yutaka Yasui, Kevin C. Oeffinger, Claudia E. Kuehni

**Affiliations:** ^1^ Childhood Cancer Research Group, Institute of Social and Preventive Medicine University of Bern Bern Switzerland; ^2^ Center for Primary Care and Public Health (Unisanté) University of Lausanne Lausanne Switzerland; ^3^ Division of Pediatric Oncology/Hematology, University Children's Hospital Basel University of Basel Basel Switzerland; ^4^ Division of Pediatric Oncology and Hematology, Department of Women, Child and Adolescent, University Geneva Hospitals, Cansearch Research platform for pediatric oncology and hematology, Faculty of Medicine, Department of Pediatrics, Gynecology and Obstetrics University of Geneva Geneva Switzerland; ^5^ Department of Epidemiology and Cancer Control St. Jude Children's Research Hospital Tennessee Memphis USA; ^6^ Pediatric Hematology‐Oncology Unit University Hospital (CHUV) Lausanne Switzerland; ^7^ Department of Radiation Physics The University of Texas MD Anderson Cancer Center Houston Texas USA; ^8^ Fred Hutchinson Cancer Center Washington Seattle USA; ^9^ Aflac Cancer Center Children's Healthcare of Atlanta/Emory University Atlanta Georgia USA; ^10^ Division of Pediatric Hematology and Oncology, University Children's Hospital Bern University of Bern Bern Switzerland; ^11^ Division of Cancer Control and Population Sciences National Cancer Institute Rockville Maryland USA; ^12^ Department of Medicine Duke University and Duke Cancer Institute Durham North Carolina USA

**Keywords:** acute lymphoblastic leukemia, adiposity, cardiometabolic, childhood cancer survivors, late effect

## Abstract

**Background:**

After childhood acute lymphoblastic leukemia (ALL), sequelae include overweight and obesity, yet with conflicting evidence. We compared the prevalence of overweight and obesity between ≥5‐year ALL survivors from the North American Childhood Cancer Survivor Study (CCSS) and the Swiss Childhood Cancer Survivor Study (SCCSS) and described risk factors.

**Methods:**

We included adult childhood ALL survivors diagnosed between 1976 and 1999. We matched CCSS participants (3:1) to SCCSS participants by sex and attained age. We calculated body mass index (BMI) from self‐reported height and weight for 1287 CCSS and 429 SCCSS participants; we then compared those with siblings (2034) in North America and Switzerland (678) siblings. We assessed risk factors for overweight (BMI 25–29.9 kg/m^2^) and obesity (≥30 kg/m^2^) using multinomial regression.

**Results:**

We found overweight and obesity significantly more common among survivors in North America when compared with survivors in Switzerland [overweight: 30%, 95% confidence interval (CI): 27–32 vs. 24%, 21–29; obesity: 29%, 27–32 vs. 7%, 5–10] and siblings (overweight: 30%, 27–32 vs. 25%, 22–29; obesity: 24%, 22–26 vs. 6%, 4–8). Survivors in North America [odds ratio (OR) = 1.24, 1.01–1.53] and Switzerland (1.27, 0.74–2.21) were slightly more often obese than siblings. Among survivors, risk factors for obesity included residency in North America (5.8, 3.7–9.0); male (1.7, 1.3–2.3); attained age (≥45 years: 5.1, 2.4–10.8); Non‐Hispanic Black (3.4, 1.6–7.0); low household income (2.3, 1.4–3.5); young age at diagnosis (1.6, 1.1–2.2). Cranial radiotherapy ≥18 Gray was only a risk factor for overweight (1.4, 1.0–1.8); steroids were not associated with overweight or obesity. Interaction tests found no evidence of difference in risk factors between cohorts.

**Conclusions:**

Although treatment‐related risk for overweight and obesity were similar between regions, higher prevalence among survivors in North America identifies important sociodemographic drivers for informing health policy and targeted intervention trials.

## Abstract 2

Like with the general population, our study confirms obesity as a prevalent health hazard—driven mainly by sociodemographic factors—among survivors of childhood acute lymphoblastic leukemia and their siblings. Since cancer survivors are particularly susceptible to cardiovascular disease and other late sequelae of overweight and obesity, we recommend a two‐pronged approach: health policies for reducing overweight among the general population and interventions targeting physical activity and diet during and after cancer treatment.

## 1 INTRODUCTION

Acute lymphoblastic leukemia (ALL) is the most common childhood cancer, comprising 25% of all childhood cancer diagnoses.^1^ Survival has improved in many high‐income countries to over 90% in recent years,^2^ resulting in a growing population of childhood ALL survivors at risk of late effects.^3^, ^4^ Among these, overweight and obesity are of particular concern since they potentiate the risk for cardiovascular disease^5^—the most common non‐malignant cause of death among childhood cancer survivors.^6^ Prevalence of overweight and obesity varies across the world with previous studies showing 26% of survivors in Switzerland and 46% of survivors in North America of childhood ALL with overweight or obesity 15 years after ALL treatment.^7^, ^8^, ^9^, ^10^, ^11^ Cranial radiation therapy (CRT) has been described as a risk factor for obesity in North America^8^, ^9^, ^12^, ^13^, ^14^ and Switzerland,^7^, ^15^ particularly among females treated at young age (0–4 years).

Risk of overweight or obesity among survivors of childhood ALL vary by inclusion criteria, comparison groups, study designs, and adjustment for risk factors.^7^, ^8^, ^9^, ^12^, ^13^, ^14^, ^16^, ^17^ Therefore, comparison across studies and countries is complex. We hypothesize geographically specific factors driving overweight and obesity, necessitating different strategies to identify survivors at risk for weight problems and introduce interventions early in follow‐up care. Therefore, we analyzed linked data from the North American Childhood Cancer Survivor Study (CCSS) and the Swiss Childhood Cancer Survivor Study (SCCSS) to determine whether the prevalence of overweight or obesity differs and to identify geographically specific risk factors for overweight and obesity.

## 2 MATERIALS AND METHODS

### 2.1 Study design and population

CCSS and SCCSS are retrospective cohort studies with questionnaire‐based longitudinal follow‐up of ≥5‐year survivors of childhood cancer diagnosed before age 21 with leukemia, lymphoma, central nervous system tumors, and malignant solid tumors. Questionnaires from CCSS (https://ccss.stjude.org) and SCCSS (https://www.swiss‐ccss.ch) include similar questions about health outcomes and explanatory variables.^18^, ^19^

CCSS is a study of 31 institutions in the United States and Canada of 5‐year survivors of childhood cancer diagnosed between 1970 and 1999.^19^ For our analyses, we included survivors of childhood ALL diagnosed between 1976 and 1999, aged ≥18 at time of follow‐up who completed either baseline (1992–2001), expansion baseline (2002–2017), follow‐up 2 (2001–2005), follow‐up 4 (2007–2009), or follow‐up 5 (2014–2016) questionnaires who provided informed consent. Although we refer to CCSS as a North American cohort, for our analysis survivors from Canada represented only 3% (44/1287). However, since the prevalence of overweight and obesity was similar between survivors in the United States and Canada, we did not exclude survivors in Canada (Table S1). CCSS is registered at ClincialTrials.gov (identifier: NCT01120353) and approved by relevant institutional review boards. We list participating institutions in Supplemental Methods.

SCCSS is a population‐based study of all children diagnosed with cancer in Switzerland. All children are treated in one of nine pediatric oncology‐hematology centers and registered in the Swiss Childhood Cancer Registry (www.childhoodcancerregistry.ch).^18^ The registry includes all children and adolescents diagnosed with cancer prior to age <21 years in Switzerland since 1976.^20^ It has a high case ascertainment of >95% for individuals diagnosed younger than age 16.^21^ We included ALL survivors diagnosed between 1976 and 1999, aged ≥18 at the time of follow‐up with completed baseline 1 (2007–2013), baseline 2 (2015–2016), or follow‐up 1 (2017) questionnaires who provided informed consent. SCCSS is registered at ClincialTrials.gov (identifier: NCT03297034). Ethical approval was granted by the ethics committee of the canton of Bern, Switzerland (KEK‐BE: 166/2014 and 2021‐01462). We list participating institutions in Supplemental Methods.

### 2.2 Sibling comparison group

We used siblings as a comparison group. During CCSS and SCCSS baseline questionnaire collection, survivor participants were asked for consent to contact siblings and their contact information. For CCSS, a random selection of nearest‐age siblings were identified. For SCCSS, all siblings of survivor participants were contacted. Siblings received the same questionnaires as survivors without questions about cancer history.^18^, ^19^

### 2.3 Outcome

We collected self‐reported body weight without clothes and height without shoes from the most recent questionnaire and calculated body mass index (BMI) by dividing weight by height in meters squared (kg/m^2^). We used BMI as a continuous and categorical variable with the following cutoffs: underweight (<18.5 kg/m^2^); normal weight (18.5–24.9 kg/m^2^); overweight (25–29.9 kg/m^2^); obesity (≥30 kg/m^2^).^22^

### 2.4 Explanatory variables

We collected sociodemographic, socioeconomic, and lifestyle characteristics from questionnaires at the same time of the most recent BMI assessments. Sociodemographic characteristics included: sex (female, male); attained age at questionnaire (years); survey calendar year (2000–2006, 2007–2012, 2013–2017); ethnicity (non‐Hispanic White, non‐Hispanic Black, Hispanic, Asian or Pacific Islander, other, missing); living situation (alone, other, missing); education level (highest obtained degree: lower, college, missing); yearly household income (*low* CCSS baseline: <$20,000; follow‐up questionnaires: <$40,000; SCCSS: ≤54,000 Swiss francs; *middle* CCSS baseline: $20,000–60,000; follow‐up questionnaires: $40,000–100,000; and SCCSS: 54,000–108,000 Swiss francs; *high* CCSS baseline: >$60,000; follow‐up questionnaires: >$100,000; SCCSS: >108,000 Swiss francs; *missing*); smoking status (never, former, current, missing); alcohol consumption (never/rarely: <1 standard drink/week; weekly: ≥1 standard drink/week; daily: 1 standard drink/day; frequently: >1 standard drink/day; missing), and physical activity (inactive, active, missing).

We dichotomized physical activity according to the World Health Organization guidelines for adults as either meeting physical activity guidelines (≥150 min of moderate‐intense or ≥75 min of vigorous‐intense physical activity/week, or a combination of moderate and vigorous intensity physical activity per week) or not meeting physical activity guidelines (<150 min of moderate‐intense physical activity/week).^23^ Clinical characteristics included age at diagnosis (years), year of diagnosis (1976–1980; 1981–1985; 1986–1990; 1991–1995; 1996–1999), time since diagnosis (years), glucocorticoid treatment (prednisone, dexamethasone, both, none, missing), CRT (no, <18 Gray [Gy], ≥18 Gy, missing), total body irradiation (TBI; no, yes, missing), hematopoietic stem cell transplantation (HSCT; no, yes, missing), relapse (no, yes), and second malignancies (no, yes, missing).

### 2.5 Statistical analysis

We weighted siblings so they became representative of survivors regarding the distribution of key sociodemographic variables (sex, attained age, and ethnicity). We fitted a logistic regression with survivorship status (survivor vs. sibling) as the outcome and the key sociodemographic variables as predictors. We calculated analysis weights for siblings as the inverse probability of being a survivor estimated from this regression. We matched ALL survivors in North America and Switzerland based on sex (exact) and attained age (±2 years) on a 3:1 ratio. We matched siblings in North America with siblings in Switzerland in the same way. We analyzed CCSS and SCCSS datasets separately for the comparison between ALL survivors and siblings. We handled missing values with multiple imputation by chained equations assuming missing at random,^24^ generating 10 imputed datasets, and pooling the results according to the Rubin's rules.^25^

We used univariable and multivariable multinomial logistic regressions (BMI categories) to identify factors associated with overweight and obesity in the pooled CCSS and SCCSS datasets and performed interaction tests to see whether effect estimates of risk factors differed between cohorts. Since BMI is a continuous rather than categorical trait, we ran sensitivity analyses investigating factors associated with BMI in a multivariable linear regression (BMI continuous). We used STATA software (version 16, Stata Corporation) for all analyses.

## 3 RESULTS

### 3.1 Study population

We included 1287 ALL survivors in North America (1243 United States; 44 Canada) and 429 from Switzerland; and 2034 siblings in North America and 678 in Switzerland (Figure S1). The mean attained age was 30.5 years (standard deviation [SD] 7.6 years) for ALL survivors of both cohorts after matching (Table 1; Tables S2 and S3). The mean age at diagnosis was 7.5 years (SD 4.8) for participants in North America and 6.2 years (SD 4.0) for participants from Switzerland (Table 2). More participants from North America reported receiving CRT ≥18 Gy than from Switzerland (38% vs. 14%).

**TABLE 1:** Demographic, socioeconomic, and lifestyle characteristics of ALL survivors and siblings comparing CCSS (North America) with SCCSS (Switzerland).

Characteristics	ALL survivors				Siblings^a^			
	CCSS (North America)^b^ *n* = 1287		SCCSS (Switzerland) *n* = 429		CCSS (North America)^b^ *n* = 2034		SCCSS (Switzerland) *n* = 678	
	*n*	%	*n*	%	*n*	%_std_	*n*	%_std_
Sex								
Female	663	52	221	52	1206	52	402	52
Male	624	48	208	48	828	48	276	48
Attained age (years)								
Mean (SD)	30.5	7.6	30.5	7.6	30.8	7.3	30.6	8.2
<25	355	28	118	28	443	28	234	27
25–34	553	43	186	43	969	43	281	43
35–44	330	26	109	25	540	25	136	26
≥45	49	4	16	4	82	4	27	4
Calendar year of survey								
2000–2006	240	19	0	0	745	18	0	0
2007–2012	313	24	195	45	299	22	678	100
2013–2017	734	57	234	55	990	59	0	0
Ethnicity								
Non‐Hispanic White	1013	79	415	97	1745	79	669	96
Non‐Hispanic Black	54	4	‐	‐	68	4	‐	‐
Hispanic	165	13	9	2	97	12	4	2
Asian or Pacific Islander	26	2	1	<1	27	2	1	<1
Other	27	2	‐	‐	37	2	‐	‐
*Missing*	*2*	<1	*4*	*<1*	*60*	*<1*	*4*	*<1*
Living situation								
Alone	95	7	81	19	127	7	97	15
Other	696	54	347	81	1098	62	575	85
*Missing*	*496*	*39*	*1*	*<1*	*809*	*31*	*6*	*<1*
Education level (highest degree)^c^								
Lower	756	59	338	79	1064	52	316	46
College	514	40	91	21	892	44	354	53
*Missing*	*17*	*1*	*‐*	*‐*	*78*	*4*	*8*	1
Household income^d^								
Low	371	29	102	24	441	22	85	12
Middle	435	34	182	42	814	40	259	38
High	210	16	57	13	507	25	161	26
*Missing*	271	*21*	88	21	*272*	*13*	*173*	*24*
Smoking status								
Never	860	67	250	58	1172	61	416	60
Former	218	17	72	17	427	20	125	20
Current	179	14	104	24	408	18	130	20
*Missing*	*30*	*2*	*3*	*<1*	*27*	*1*	*7*	*1*
Alcohol consumption^e^								
Never/rarely	751	58	207	48	1107	56	357	52
Weekly	196	15	200	47	396	18	281	41
Daily	6	<1	10	2	31	<1	20	4
Frequently	19	1	5	1	104	3	18	3
*Missing*	*315*	*24*	*7*	*2*	*396*	*22*	*2*	<1
Physical activity^f^								
Inactive	276	21	84	20	357	18	108	16
Active	368	29	327	76	668	34	560	82
*Missing*	*643*	*50*	*18*	*4*	*1009*	*47*	*10*	*2*
BMI (kg/m^2^)								
Mean (SD)	27.4	6.5	23.6	4.0	26.7	7.0	23.8	4.0
Underweight, <18.5	39	3	29	7	64	3	12	1
Normal, 18.5–24.9	489	38	265	62	902	44	477	68
Overweight, 25–29.9	384	30	105	24	593	30	152	25
Obese, ≥30	375	29	30	7	475	24	37	6

Abbreviations: ALL, acute lymphoblastic leukemia; BMI, body mass index; CCSS, Childhood Cancer Survivors Study; IQR, interquartile range; SCCSS, Swiss Childhood Cancer Survivors Study; SD, standard deviation; sd, standardized.

The italic values give insights about the number of missing values for each characteristic, country, and population group (survivors vs siblings).

^a^ Siblings standardized by sex, attained age, and race/ethnicity to ALL survivors by cohort.

^b^ We matched ALL survivors/siblings from North America with survivors/siblings in Switzerland on a 1:3 ratio based on sex and attained age.

^c^ Highest degree of education level is categorized as lower than college graduate/post graduate level and college graduate/post graduate level.

^d^ Household income (income per year) is categorized as *low*: CCSS baseline (1992–2001): <$20,000, expansion baseline (2002–2017), follow‐up 2 (2001–2005), follow‐up 4 (2007–2009), and follow‐up 5 (2014–2016): <$40,000, SCCSS: ≤54,000 Swiss francs; *middle*: CCSS: baseline: $20,000–60,000, other questionnaires: $40,000–100,000, SCCSS: 54,000–108.000 Swiss francs; and *high*: CCSS baseline: >$60,000, other questionnaires: >$100,000, SCCSS: >108,000 Swiss francs.

^e^ Alcohol consumption is categorized as never/rarely; weekly, ≥1 standard drink/week; daily, 1 standard drink/day; frequently, >1 standard drink/day.

^f^ *Physically inactive* is defined as fewer than 150 min of activity per week; *physically active* is defined as 150 min or more of moderate or 75 min of vigorous physical activity, or a combination of moderate and vigorous‐intense physical activity per week.

**TABLE 2:** Clinical (diagnosis and treatment) characteristics of ALL survivors comparing CCSS (North America) with SCCSS (Switzerland).

Characteristics	ALL survivors			
	CCSS^a^ (North America) *n* = 1287		SCCSS (Switzerland) *n* = 429	
	*n*	*%*	*n*	*%*
Age at diagnosis (years)				
Mean (SD)	7.5	4.8	6.2	4.0
Median (IQR)	6.1	(3.6; 11.2)	5.0	(3.1; 8.7)
<5	528	41	214	50
5–9	368	29	132	31
≥10	391	30	83	19
Year of diagnosis				
1976–1980	316	25	57	13
1981–1985	221	17	88	21
1986–1990	249	19	110	26
1991–1995	307	24	118	28
1996–1999	194	15	56	13
Time since diagnosis				
Mean (SD)	23.2	6.9	24.3	7.2
Median (IQR)	22.6	(18.3; 27.6)	23.5	(19.0; 29.5)
<10	23	2	3	1
11–14	108	8	37	9
15–19	309	24	96	22
≥20	847	66	293	68
Glucocorticoids				
Prednisone	768	60	295	69
Dexamethasone	43	3	1	<1
Both	318	25	115	27
None	46	4	16	4
*Missing*	*112*	*9*	*2*	<1
CRT (gray)				
No	630	49	330	77
<18	26	2	37	9
≥18	492	38	62	14
*Missing*	*139*	*11*	‐	‐
Total body radiation				
No	1115	87	422	98
Yes	35	3	7	2
*Missing*	*138*	*11*	‐	‐
HSCT				
No	1116	87	413	96
Yes	54	4	16	4
*Missing*	*117*	*9*	‐	‐
Relapse				
No	1156	90	375	87
Yes	131	10	54	13
Second malignancies				
No	1252	97	400	93
Yes	35	3	15	4
*Missing*	‐	‐	*14*	*3*

Abbreviations: ALL, acute lymphoblastic leukemia; BMI, body mass index; CCSS, Childhood Cancer Survivor Study; CRT, cranial radiation therapy; HSCT, hematopoietic stem cell transplantation; IQR, interquartile range; SCCSS, Swiss Childhood Cancer Survivor Study; SD, standard deviation.

^a^ We matched ALL survivors in North America with ALL survivors in Switzerland on a 1:3 ratio based on sex and attained age.

### 3.2 Overweight and obesity

**FIGURE 1:**
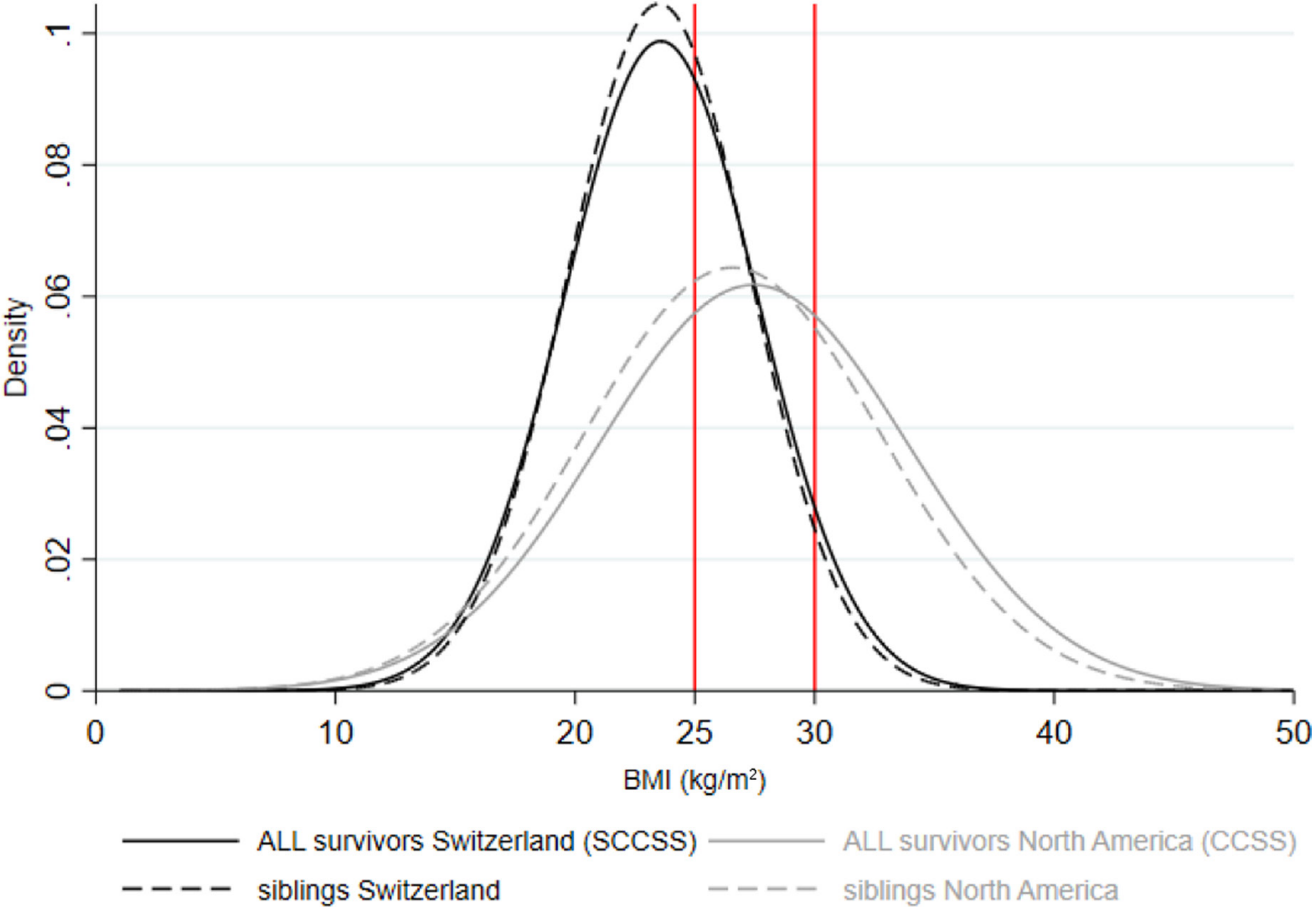
Distribution of body mass index (BMI) for ALL survivors and siblings in the CCSS (North America) and the SCCSS (Switzerland).^a,b^ CCSS, Childhood Cancer Survivor Study; SCCSS, Swiss Childhood Cancer Survivor Study. ^a^We matched ALL survivors/siblings in North America with survivors/siblings in Switzerland on a 3:1 ratio based on sex and attained age. ^b^Siblings are standardized by sex, attained age, and race/ethnicity to ALL survivors by cohort.

We identified more ALL survivors from North America with overweight than Switzerland [30%, 95% confidence interval (CI) 27–32 vs. 24%, 95% CI 21–29] and obesity (29%, 95% CI 27–32 vs. 7%, 95% CI 5–10) (Table 1; Figure 1). We found the same for siblings (overweight: 30%, 95% CI 27–32 vs. 25%, 95% CI 22–29; obesity: 24%, 95% CI 22–26 vs. 6%, 95% CI 4–8). When adjusting for demographic, socioeconomic, and lifestyle factors, we found a similar magnitude of increased risks for obesity among survivors in North America compared with their siblings [odds ratio (OR) = 1.24; 95% CI: 1.01–1.53] and for survivors in Switzerland compared with their siblings (OR = 1.27; 95% CI: 0.74–2.21) (Table 3).

**TABLE 3:** Overweight and obesity among ALL survivors compared with siblings from CCSS (North America) and SCCSS (Switzerland) (referent: normal BMI): a multinomial logistic regression analysis.

	Overweight vs. Normal^a^				Obesity vs. Normal^a^			
	OR (95% CI)				OR (95% CI)			
	*n*_ow_/*n*_nor_	Unadjusted	Model 1^b^	Model 2^c^	*n*_ob_/*n*_nor_	Unadjusted	Model 1^b^	Model 2^c^
CCSS (North America)								
Siblings^d^	593/902	1.00 (ref)	1.00 (ref)	1.00 (ref)	475/902	1.00 (ref)	1.00 (ref)	1.00 (ref)
ALL survivors	384/489	1.15 (0.96; 1.38)	1.18 (0.98; 1.43)	1.12 (0.92; 1.36)	375/489	1.40 (1.16; 1.70)	1.46 (1.20; 1.77)	1.24 (1.01; 1.53)
SCCSS (Switzerland)								
Siblings^d^	152/477	1.00 (ref)	1.00 (ref)	1.00 (ref)	37/477	1.00 (ref)	1.00 (ref)	1.00 (ref)
ALL survivors	105/265	1.08 (0.81; 1.45)	1.05 (0.77; 1.43)	1.02 (0.75; 1.40)	30/265	1.33 (0.80; 2.22)	1.33 (0.79; 2.24)	1.27 (0.74; 2.21)

Abbreviations: ALL, acute lymphoblastic leukemia; BMI, body mass index; CCSS, Childhood Cancer Survivor Study; nor, normal; ob, obese; OR, odds ratio, ow, overweight; SCCSS, Swiss Childhood Cancer Survivor Study.

^a^ We excluded those with underweight: ALL survivors in North America: 39; ALL survivors in Switzerland: 29; siblings in North America: 64; siblings in Switzerland: 12.

^b^ Model 1 is adjusted for sex and attained age.

^c^ Model 2 is adjusted for sex, attained age, race/ethnicity, household income, smoking status, alcohol consumption, and physical activity.

^d^ Siblings are standardized by sex, attained age, and race/ethnicity to ALL survivors by cohort.

### 3.3 Predictors for overweight and obesity

In univariable and multivariable logistic regressions of pooled datasets adjusted for demographic, socioeconomic, lifestyle, and clinical factors, we found predictors for overweight and/or obesity were residency in North America compared with Switzerland (OR_overweight_ = 1.71; 95% CI: 1.25–2.34; OR_obesity_ = 5.78; 95% CI: 3.70–9.03); male compared with female (OR_overweight_ = 2.04; 95% CI: 1.59–2.61; OR_obesity_ = 1.73; 95% CI: 1.31–2.28); older attained age compared with younger attained age (≥45 years, OR_obesity_ = 5.12; 95% CI: 2.42–10.83); non‐Hispanic Black ethnicity (OR_obesity_ = 3.38; 95% CI: 1.62–7.04) or Hispanic compared with Non‐Hispanic White ethnicity (OR_obesity_ = 1.66; 95% CI: 1.08–2.54); low household income compared with high household income (OR_obesity_ = 2.25; 95% CI: 1.43–3.54), formerly smoking compared with never smoking before (OR_overweight_ = 1.52; 95% CI: 1.10–2.10). We detected trends for not meeting physical activity guidelines (OR_obesity_ = 1.39; 95% CI: 1.00–1.94); age <5 years compared with age ≥10 years at diagnosis (OR_obesity_ = 1.55; 95% CI:1.08–2.23); CRT ≥18 Gy treatment compared with no CRT (OR_overweight_ = 1.38; 95% CI: 1.04–1.83). Steroids were not associated with overweight/obesity (Table 4 for multivariable, Table S5 for univariable results).

**TABLE 4:** Predictors for overweight and obesity among ALL survivors (retrieved from multivariable multinomial logistic regression^a^; referent: normal BMI).

Characteristics	Overweight vs. Normal		Obesity vs. Normal	
	OR (95% CI)	*p*	OR (95% CI)	*p*
Cohort				
SCCSS (Switzerland)	1.00 (ref)	0.001	1.00 (ref)	<0.001
CCSS (North America)^b^	1.71 (1.25; 2.34)		5.78 (3.70; 9.03)	
Sex				
Female	1.00 (ref)	<0.001	1.00 (ref)	<0.001
Male	2.04 (1.59; 2.61)		1.73 (1.31; 2.28)	
Attained age, years				
<25	1.00 (ref)	<0.001	1.00 (ref)	<0.001
25–34	1.55 (1.14; 2.11)		2.01 (1.40; 2.86)	
35–44	2.12 (1.47; 3.06)		3.48 (2.29; 5.30)	
≥45	1.41 (0.67; 2.95)		5.12 (2.42; 10.83)	
Ethnicity				
Non‐Hispanic White	1.00 (ref)	0.224	1.00 (ref)	0.004
Non‐Hispanic Black	2.00 (0.91; 4.38)		3.38 (1.62; 7.04)	
Hispanic	1.39 (0.91; 2.11)		1.66 (1.08; 2.54)	
Asian or Pacific Islander	1.18 (0.45; 3.08)		1.01 (0.35; 2.94)	
Other	1.59 (0.64; 3.93)		0.71 (0.23; 2.22)	
Household income^c^				
Low	1.45 (1.99; 2.11)	0.195	2.25 (1.43; 3.54)	<0.001
Middle	1.24 (0.86; 1.78)		1.47 (0.96; 2.27)	
High	1.00 (ref)		1.00 (ref)	
Smoking status				
Never	1.00 (ref)	0.009	1.00 (ref)	0.702
Former	1.52 (1.10; 2.10)		1.17 (0.81; 1.69)	
Current	0.81 (0.57; 1.15)		0.99 (0.67; 1.46)	
Alcohol^d^				
Never/rarely	1.00 (ref)	0.774	1.00 (ref)	0.149
Weekly	0.85 (0.61; 1.20)		0.66 (0.45; 0.97)	
Daily	0.81 (0.24; 2.75)		0.77 (0.17; 3.53)	
Frequently	0.78 (0.30; 2.01)		0.51 (0.14; 1.84)	
Physical activity^e^				
Inactive	1.19 (0.81; 1.75)	0.353	1.39 (1.00; 1.94)	0.050
Active	1.00 (ref)		1.00 (ref)	
Age at diagnosis (years)				
<5	1.00 (0.72; 1.38)	0.944	1.55 (1.08; 2.23)	0.042
5–9	0.95 (0.69; 1.32)		1.18 (0.782; 1.70)	
≥10	1.00 (ref)		1.00 (ref)	
CRT (gray)				
No	1.00 (ref)	0.088	1.00 (ref)	0.323
<18	1.09 (0.69; 1.74)		1.16 (0.69; 1.696)	
≥18	1.38 (1.04; 1.83)		1.26 (0.93; 1.672)	
Total body radiation				
No	1.00 (ref)	0.043	1.00 (ref)	0.003
Yes	0.39 (0.16; 0.97)		0.21 (0.07; 0.59)	

Abbreviations: ALL, acute lymphoblastic leukemia; BMI, body mass index; CCSS, Childhood Cancer Survivor Study; CRT, cranial radiation therapy; HSCT, hematopoietic stem cell transplantation; OR, odds ratio; ref, reference; SCCSS, Swiss Childhood Cancer Survivor Study.

^a^ Adjusted for all variables listed.

^b^ We matched ALL survivors in North America with survivors in Switzerland on a 1:3 ratio based on sex and attained age.

^c^ Household income (income per year) is categorized as *low*: CCSS baseline (1992–2001): <$20,000, expansion baseline (2002–2017), follow‐up 2 (2001–2005), follow‐up 4 (2007–2009), and follow‐up 5 (2014–2016): <$40,000, SCCSS: ≤54,000 Swiss francs; *middle*: CCSS: baseline: $20,000–60,000, other questionnaires: $40,000–100,000, SCCSS: 54,000–108,000 Swiss francs, and *high*: CCSS baseline: >$60,000, other questionnaires: >$100,000, SCCSS: >108,000 Swiss francs.

^d^ Alcohol consumption is categorized as never/rarely; weekly, ≥1 standard drink/week; daily, 1 standard drink/day; frequently, >1 standard drink/day.

^e^ *Physically inactive* is defined as fewer than 150 min of activity per week; *physical active* is defined as 150 min or more of moderate or 75 min of vigorous physical activity, or a combination of moderate and vigorous‐intense physical activity per week.

In the two cohorts, we found associations with demographic, socioeconomic, and clinical factors followed the same direction and of comparable strength (all *p*‐values for interactions ≥0.05), suggesting drivers of obesity are the same in North America and Switzerland (Table S8). The only difference was the association with CRT, which was weaker in North America (OR = 1.14, 95% CI: 0.82–1.58) than in Switzerland (OR = 3.10, 95% CI: 1.08–8.89) (Table S6). The number of survivors with obesity who received ≥18 Gy CRT was small (11 of 30 in Switzerland and 156 out of 375 in North America (Table S4).

The multivariable linear regression models—which modeled BMI as a continuous outcome—identified the same predictors as the logistic regression models. In fact, associations were stronger, in particular for young age at diagnosis, physical activity, and CRT (Table S7).

## 4 DISCUSSION

Our collaborative analysis found overweight and obesity more common among survivors of childhood ALL and their siblings in North America than Switzerland. Although our results demonstrate an increased risk for obesity among survivors compared with siblings, the main risk factors for overweight and obesity in both cohorts were sociodemographic—not treatment‐related—factors.

To our knowledge, our study is the first direct comparison of adult survivors of childhood ALL involving high‐income countries across two continents, yet with differences regarding lifestyle and socioeconomic risk factors within each society.^26^, ^27^, ^28^, ^29^, ^30^ Our findings contribute to understanding the differential development of obesity globally after ALL treatment during childhood. First, we found evidence of a treatment exposure role. CRT (≥18 Gy) was significantly associated with overweight, consistent with previous separate analyses of both cohorts^7^, ^8^, ^9^, ^15^ and other studies.^13^, ^14^, ^17^ However, such finding is largely of historical interest since CRT is rarely used in contemporary treatment of ALL, and the effect size was relatively small. Second, we found risk for obesity was higher among children diagnosed and treated for ALL at young age (0–4 years). It is possible chemotherapy and radiotherapy effects on growing bodies are stronger during this developmental window. Alternatively—and perhaps more likely—the long, intensive treatment possibly disturbs the development of individual patterns of physical activity occurring at this age. For two or more years, children diagnosed with ALL are repeatedly hospitalized, receive chemotherapy, and experience restrictions of social contacts to reduce the risk of infection. So, opportunities to socialize, play, and run around with peers are limited and parents may be overprotective; it is possibly more relevant during preschool when activity patterns are consolidated.^31^, ^32^ Physical activity habits acquired during early childhood strongly track throughout life.^33^ For children diagnosed later, it might be easier to return to previous physical activity and lifestyle habits after treatment ends, while children diagnosed when preschoolers likely do not remember a physical activity period from before. In our study, we found no evidence that treatment with steroids was a strong predictor of overweight and obesity in long‐term survivors of childhood ALL, as shown before within the SCCSS.^15^ During and shortly after treatment, exposure to steroids can lead to overweight and obesity, but in the long‐term we found that sociodemographic factors seem to play a more important role. However, within our study, we were not able to compare different doses of steroids, so we cannot exclude effects of higher doses of steroids.

The dramatic differences between the prevalence of obesity between participants from North America—survivors and siblings—and participants from Switzerland shows how strongly lifestyle factors, such as diet and physical activity, influence overall risk. In the United States general population, about 30% of adults aged 20–39 are obese^34^; in Switzerland, the proportion is 11%.^7^, ^35^ Our findings for survivors with obesity—29% in North America and 7% in Switzerland—is comparable. It means the risk of survivors developing obesity is not an unavoidable result of cancer treatment. Instead, it reflects the general population and suggests it is potentially avoidable with health policy changes and early lifestyle interventions at the population level. In fact, our study shows household income and ethnicity—socioeconomic factors affecting physical activity and diet—with a much stronger impact on overweight than cancer treatment when comparing survivors with siblings, which was consistent across cohorts.

The associations found with age reflect findings from the general population.^34^, ^35^ In both cohorts, males showed higher risk for overweight and obesity than females. Although it contrasts with previous CCSS publications,^8^, ^9^, ^11^, ^12^, ^36^ it aligns with an SCCSS study^7^ and findings from general Swiss and Non‐Hispanic, White US populations.^34^, ^35^ A meta‐analysis of 10 studies from the United States, The Netherlands, and the United Kingdom found no risk difference by sex.^10^ Thus the higher risk for males with ALL likely reflects the higher background risk in the general population of Switzerland and North America.^34^, ^35^

The comparability of the two cohorts demonstrates the main strength of our study, which share the same design, methodology, and questionnaires. Inclusion criteria—era of cancer diagnosis (1976–1999), sex, and attained age—were identical and prevented biases and difficulties other studies encountered when interpreting results. Our study also has limitations. Since participants self‐reported height and weight in both cohorts, survivors possibly underestimated weight or overestimated height due to social desirability bias. However, we expect the degree of error of BMI assessment to be non‐differential, that is, similar for survivors and siblings in both cohorts. Cumulative doses of CRT were assessed by an intention‐to‐treat approach for SCCSS and by detailed dosimetry in CCSS.^37^ Thus, the measurement error for CRT was larger for survivors in Switzerland, which possibly attenuated effect estimates. Furthermore, we could not investigate risk factors for overweight since both cohorts lacked information, namely physical functioning, BMI before or at diagnosis, parental BMI, and genetic factors.^13^, ^14^, ^38^

## 5 CONCLUSIONS

Overall, our study demonstrated overweight, and obesity are highly prevalent and thus a major health concern with nearly two thirds of childhood ALL survivors in North America and one third in Switzerland with overweight or obesity. It is established obesity potentiates the risk for cardiovascular disease,^5^ which is deleterious for childhood ALL survivors with already high burdens of cardiovascular disease and related mortality from cancer and treatment with cardiotoxic anthracycline chemotherapy.^6^, ^39^ We recommend identifying survivors at risk for cardiovascular disease early and offering support with targeted interventions.^40^, ^41^ Physical activity has been shown as beneficial and safe during and after treatment,^42^, ^43^ yet currently well below recommended levels.^44^, ^45^, ^46^, ^47^ Some children need special support because of musculoskeletal or medical impairments.^48^, ^49^ Our findings further suggest that introduction of physical activity—as a regular supportive treatment during acute cancer treatment—should be considered as interventions for evaluation among preschoolers since activity patterns acquired early track during the lifetime.^31^, ^32^ The German Network ActiveOncoKids implements such a physical activity program as usual care for pediatric and adolescent patients during and after cancer treatment. Sports scientists offer individualized trainings on a daily basis in addition to medical services, such as physiotherapy.^50^

Obesity is multi‐factorial in origin and survivors of ALL share most risk factors with the general population. Policy measures and structural changes aiming to reduce obesity in the population at large also automatically improve the situation for ALL survivors. Such measures possibly include more physical activity lessons at school, availability of safe outdoor spaces for physical activity, appropriate food labeling, access to healthy food choices, sugar taxes, close monitoring of weight trajectory and nutrition, and physical and behavioral counseling by pediatricians and general practitioners.

Our study confirmed strong effects of socioeconomic status on overweight, namely income and ethnicity. Thus, national policies to reduce social differentials in health and income in Switzerland and North America are essential for improving the situation. An international comparison of socioeconomic inequalities in adolescent health among 34 North American and European countries found inequalities between socioeconomic groups increased over time and confirmed physical activity levels and health as related to average per‐person income and income inequality within a country.^28^

Our study confirmed obesity as a prevalent health hazard among survivors of ALL and their siblings mainly driven by sociodemographic factors, like in the general population. Since cancer survivors are particularly susceptible to cardiovascular disease and other late sequelae of overweight and obesity, we recommend a two‐pronged approach: health policies for reducing overweight among the general population and interventions targeting physical activity and diet during and after cancer treatment.

## AUTHOR CONTRIBUTIONS

**Fabiën N. Belle:** Formal analysis (lead); writing – original draft (equal); writing – review and editing (equal). **Christina Schindera:** Formal analysis (supporting); writing – original draft (equal); writing – review and editing (equal). **Marc Ansari:** Writing – review and editing (supporting). **Gregory Armstrong:** Writing – review and editing (supporting). **Maja Beck‐Popovic:** Writing – review and editing (supporting). **Rebecca M. Howell:** Writing – review and editing (supporting). **Wendy M. Leisenring:** Writing – review and editing (supporting). **Lillian Meacham:** Writing – review and editing (supporting). **Jochen Rössler:** Writing – review and editing (supporting). **Ben D. Spycher:** Formal analysis (supporting); writing – review and editing (supporting). **Emily S. Tonorezos:** Writing – review and editing (supporting). **Nicolas X. von der Weid:** Writing – review and editing (supporting). **Yutaka Yasui:** Formal analysis (supporting); writing – review and editing (supporting). **Kevin C Oeffinger:** Supervision (equal); writing – original draft (supporting); writing – review and editing (supporting). **Claudia E. Kuehni:** Formal analysis (supporting); supervision (equal); writing – original draft (supporting); writing – review and editing (supporting).

## CONFLICT OF INTEREST STATEMENT

J.R. was head of Pediatric Hematology/Oncology at the University Hospital in Bern, Switzerland during the study conduct; currently he is an employee of Novartis Pharma AG. All other authors declare no competing financial interests.

## Supporting information


Data S1.

## Data Availability

The data that support the findings of this study are available from the the senior author Claudia E. Kuehni (claudia.kuehni@unibe.ch) or Kevin Oeffinger (kevin.oeffinger@duke.edu) upon reasonable request.
