# Supplementary material for: Risk factors for overweight and obesity after childhood acute lymphoblastic leukemia in North America and Switzerland: A comparison of two cohort studies
Source: Cancer Med. 2023 Oct 9;12(20):20423–36. doi: 10.1002/cam4.6588 (PMC10652345; doi:10.1002/cam4.6588)
Supplement: Supplementary file 1 — Data S1. [file CAM4-12-20423-s001.docx]

**SUPPLEMENTAL MATERIAL**

- **FIGURE S1.** Response rates in the North American Childhood Cancer Survivor Study (CCSS) and the Swiss Childhood Cancer Survivor Study (SCCSS)
- **TABLE S1.** Demographic, socio-economic, and lifestyle characteristics of CCSS ALL survivors; USA institutions versus Canada
- **TABLE S2.** Demographic, socio-economic, and lifestyle characteristics of ALL survivors by cohort and BMI category
- **TABLE S3.** Demographic, socio-economic, and lifestyle characteristics of siblings by cohort and BMI category
- **TABLE S4.** Clinical characteristics of ALL survivors by cohort and BMI category
- **TABLE S5**. Predictors for overweight and obesity in ALL survivors retrieved from univariable multinominal logistic regression; referent: normal BMI
- **TABLE S6.** Predictors for overweight and obesity in ALL survivors comparing the CCSS (North America) with the SCCSS (Switzerland) retrieved from multivariable multinominal logistic regression; referent: normal BMI
- **TABLE S7.** Predictors for overweight and obesity in ALL survivors retrieved from multivariable linear regression
- **TABLE S8. I**nteraction of cohort with demographic, socio-economic, lifestyle and clinical characteristics retrieved from multivariable multinominal logistic regression; referent: normal BMI
- **SUPPLEMENTAL METHODS:** Childhood Cancer Survivors Study institutions and Swiss Childhood Cancer Survivor Study institutions

**FIGURE S1. Response rates in the North American Childhood Cancer Survivor Study (CCSS) and the Swiss Childhood Cancer Survivor Study (SCCSS)**

**
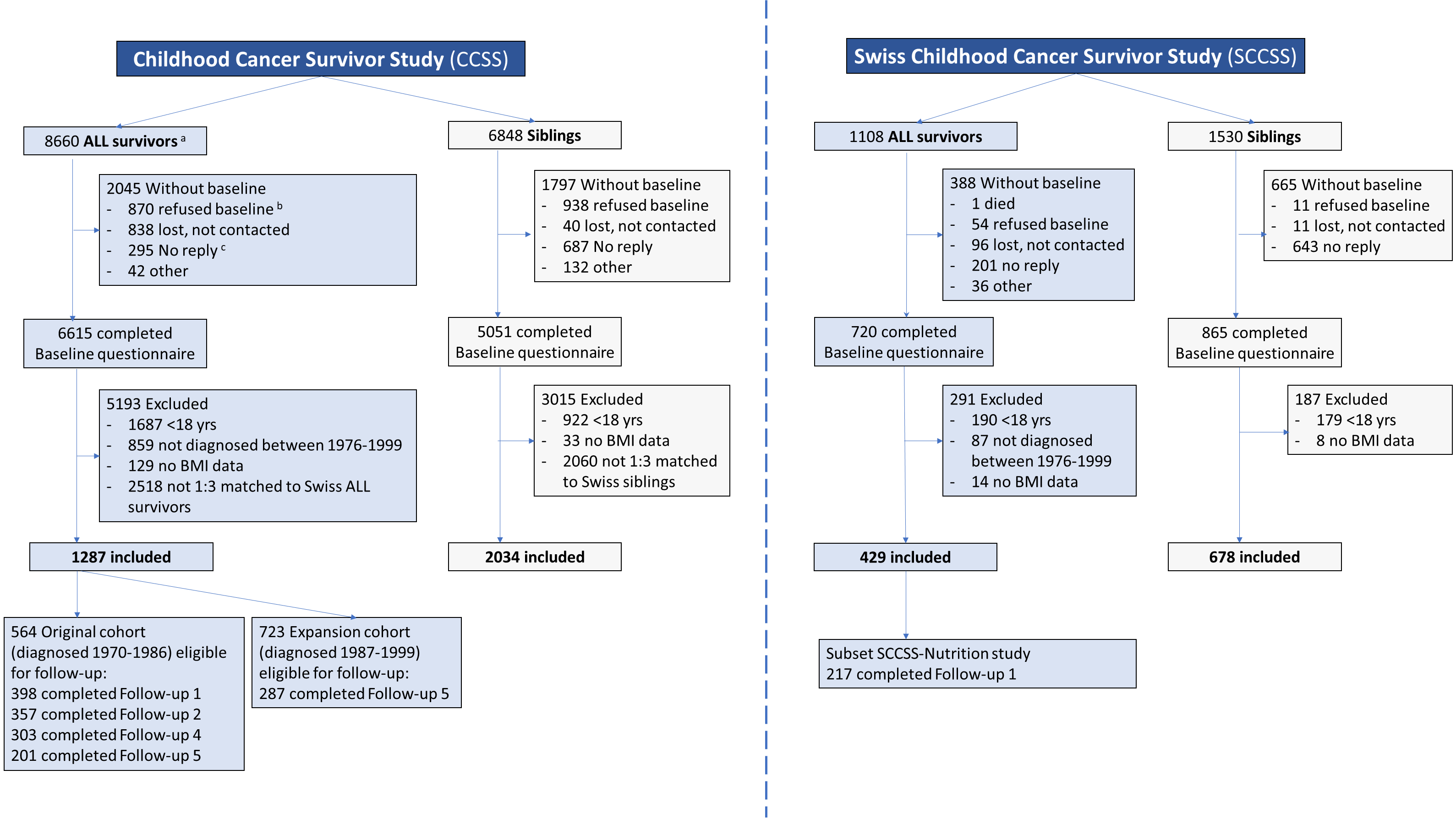
**

^a^ Out of 39209 eligible survivors, 5017 did not consent resulting in 34192 consented survivors. Of those consented, 25332 were not diagnosed with ALL, resulting in 8660 ALL survivors who were invited to complete a baseline questionnaire.

^b^ 803 passive and active refusals in the Original cohort (diagnosed 1970–1986) and 67 active refusals in the Expansion cohort (diagnosed 1987–1999).

^C^ No reply (passive refusal) information is only available for those included in the Expansion cohort (diagnosed 1987–1999).

**TABLE S1.** Demographic, socio-economic, and lifestyle characteristics of CCSS ALL survivors; USA institutions versus Canada

|  |  | **CCSS** | | | | | | |
| --- | --- | --- | --- | --- | --- | --- | --- | --- |
|  |  | **Total** | | **USA institutions** | | **Canada**^a^ | |  |
|  |  |  | |  | |  | |  |
|  |  | *n=1287* | | *n=1243* | | *n=44* | | *p^b^* |
| **Characteristics** | | *n* | *%* | *n* | *%* | *n* | *%* |  |
| **Sex** | |  |  |  |  |  |  |  |
|  | Female | 663 | 52 | 643 | 52 | 20 | 45 | 0.413 |
|  | Male | 624 | 48 | 600 | 48 | 24 | 55 |  |
| **Attained age (years)** | |  |  |  |  |  |  |  |
|  | Mean (SD) | 30.5 | 7.6 | 30.4 | 7.5 | 33.0 | 8.8 | 0.169 |
|  | <25 | 355 | 28 | 343 | 28 | 12 | 27 |  |
|  | 25–34 | 553 | 43 | 540 | 43 | 13 | 30 |  |
|  | 35–44 | 330 | 26 | 314 | 25 | 16 | 36 |  |
|  | ≥45 | 49 | 4 | 46 | 4 | 3 | 7 |  |
| **Calendar year of survey** | |  |  |  |  |  |  |  |
|  | 2000–2006 | 240 | 19 | 224 | 18 | 16 | 36 | **<0.001** |
|  | 2007–2012 | 313 | 24 | 298 | 24 | 15 | 34 |  |
|  | 2013–2017 | 734 | 57 | 721 | 58 | 13 | 30 |  |
| **Ethnicity** | |  |  |  |  |  |  |  |
|  | Non-Hispanic White | 1013 | 79 | 974 | 78 | 39 | 89 | **<0.001** |
|  | Non-Hispanic Black | 54 | 4 | 54 | 4 | - | - |  |
|  | Hispanic | 165 | 13 | 164 | 13 | 1 | 2 |  |
|  | Asian or Pacific Islander | 26 | 2 | 26 | 2 | - | - |  |
|  | Other | 27 | 2 | 24 | 2 | 3 | 7 |  |
|  | *Missing* | *2* | <1 | *1* | *<1* | *1* | *2* |  |
| **Living situation** | |  |  |  |  |  |  |  |
|  | Alone | 95 | 7 | 90 | 7 | 5 | 11 | 0.455 |
|  | Other | 696 | 54 | 671 | 54 | 25 | 57 |  |
|  | *Missing* | *496* | *39* | *482* | *39* | *14* | *32* |  |
| **Education level (highest degree)**^b^ | | |  |  |  |  |  |  |
|  | Lower | 756 | 59 | 732 | 59 | 24 | 55 | 0.752 |
|  | College | 514 | 40 | 495 | 40 | 19 | 43 |  |
|  | *Missing* | *17* | *1* | *16* | 1 | 1 | *2* |  |
| **Household income**^c^ | |  |  |  |  |  |  |  |
|  | Low | 371 | 29 | 360 | 29 | 11 | 25 | 0.353 |
|  | Middle | 435 | 34 | 424 | 34 | 11 | 25 |  |
|  | High | 210 | 16 | 200 | 16 | 10 | 23 |  |
|  | *Missing* | 271 | *21* | *259* | *21* | *12* | *27* |  |
| **Smoking status** | |  |  |  |  |  |  |  |
|  | Never | 860 | 67 | 831 | 67 | 29 | 66 | 0.997 |
|  | Former | 218 | 17 | 210 | 17 | 8 | 18 |  |
|  | Current | 179 | 14 | 173 | 14 | 6 | 14 |  |
|  | *Missing* | *30* | *2* | *29* | *2* | *1* | *2* |  |
| **Alcohol consumption**^d^ | |  |  |  |  |  |  |  |
|  | Never/rarely | 751 | 58 | 727 | 58 | 24 | 55 | 0.445 |
|  | Weekly | 196 | 15 | 190 | 15 | 6 | 14 |  |
|  | Daily | 6 | <1 | 5 | <1 | 1 | 2 |  |
|  | Frequently | 19 | 1 | 18 | 1 | 1 | 2 |  |
|  | *Missing* | *315* | *24* | *303* | 24 | *12* | *27* |  |
| **Physical activity**^e^ | |  |  |  |  |  |  |  |
|  | Inactive | 276 | 21 | 262 | 21 | 14 | 32 | **0.021** |
|  | Active | 368 | 29 | 351 | 28 | 17 | 39 |  |
|  | *Missing* | *643* | *50* | *630* | *51* | 13 | *30* |  |
| **BMI (kg/m^2^)** | |  |  |  |  |  |  |  |
|  | Mean (SD) | 27.4 | 6.5 | 27.5 | 6.5 | 26.6 | 5.2 | 0.129 |
|  | Underweight, <18.5 | 39 | 3 | 36 | 3 | 3 | 7 |  |
|  | Normal, 18.5–24.9 | 489 | 38 | 475 | 38 | 14 | 32 |  |
|  | Overweight, 25–29.9 | 384 | 30 | 366 | 29 | 18 | 41 |  |
|  | Obese, ≥30 | 375 | 29 | 366 | 29 | 9 | 20 |  |

*ALL, acute lymphoblastic leukemia; BMI, body mass index; CCSS, Childhood Cancer Survivors Study; IQR, interquartile range; SCCSS, Swiss Childhood Cancer Survivors Study; SD, standard deviation*

^a^ We matched ALL survivors from North American with survivors in Switzerland on a 1:3 ratio based on sex and attained age.

^b^ Highest degree of education level is categorized as: lower than college graduate/post graduate level and college graduate/post graduate level.

^c^ Household income (income per year) is categorized as: l*ow*: CCSS baseline (1992–2001): <$20,000, Expansion baseline (2002–2017), Follow-up 2 (2001–2005), follow-up 4 (2007–2009), and follow-up 5 (2014–2016): <$40,000, SCCSS: ≤54,000 Swiss francs; *middle*: CCSS: baseline: $20,000-60,000, other questionnaires: $40,000-100,000, SCCSS: 54,000-108,000 Swiss francs, and *high*: CCSS baseline: >$60,000, other questionnaires: >$100,000, SCCSS: >108,000 Swiss francs.

^d^ Alcohol consumption is categorized as: never/rarely; weekly, ≥1 standard drink/week; daily, 1 standard drink/day; frequently, >1 standard drink/day.

^e^ *Physically inactive* is defined as fewer than 150 min of activity per week; *physically active* is defined as 150 min or more of moderate or 75 min of vigorous physical activity, or a combination of moderate and vigorous intense physical activity per week.**TABLE S2. Demographic, socio-economic, and lifestyle characteristics of ALL survivors by cohort and BMI category**

|  |  | | | | | | **BMI** | | | | | | | | | | | | | | |
| --- | --- | --- | --- | --- | --- | --- | --- | --- | --- | --- | --- | --- | --- | --- | --- | --- | --- | --- | --- | --- | --- |
|  |  | | | | | | **ALL survivors** | | | | | | | | | | | | | | |
|  | | | | | | | **Switzerland** | | | | | | | | | **North America**^a^ | | | | | |
|  |  | | | | | | *n=429* | | | | | | | | | *n=1287* | | | | | |
|  |  | | | | | | **<25 kg/m^2^** | | | | | **25-29.9 kg/m^2^** | | **≥30 kg/m^2^** | | **<25 kg/m^2^** | | **25-29.9 kg/m^2^** | | **≥30 kg/m^2^** | |
|  |  | | | | | | *n=294* | | | | | *n=105* | | *n=30* | | *n=528* | | *n=384* | | *n=375* | |
| **Characteristics** | | | | | | | *n* | | | *%* | | *n* | *%* | *n* | *%* | *n* | *%* | *n* | *%* | *n* | *%* |
| **Sex** | | | | | | |  | | |  | |  |  |  |  |  |  |  |  |  |  |
|  | Female | | | | | | 173 | | | 59 | | 36 | 34 | 12 | 40 | 305 | 57 | 169 | 44 | 189 | 51 |
|  | Male | | | | | | 121 | | | 41 | | 69 | 66 | 18 | 60 | 223 | 42 | 215 | 56 | 186 | 50 |
| **Attained age (years)** | | | | |  | | | | |  | |  |  |  |  |  |  |  |  |  |  |
|  | Mean (SD) | | | | | | 29.4 | | | 7.2 | | 32.0 | 7.7 | 36.0 | 7.9 | 29.0 | 7.5 | 31.1 | 7.3 | 32.0 | 7.7 |
|  | Median (IQR) | | | | | | 27.9 | | | (23.8; 34.5) | | 31.4 | (26.0; 37.5) | 36.4 | (31.2; 41.6) | 27.6 | (23.3; 34.1) | 30.6 | (25.3; 36.7) | 32.0 | (25.9; 37.4) |
|  | <25 | | | | | | 94 | | | 32 | | 20 | 19 | 4 | 13 | 188 | 35 | 89 | 23 | 78 | 21 |
|  | 25–34 | | | | | | 132 | | | 45 | | 46 | 44 | 8 | 27 | 222 | 42 | 169 | 44 | 162 | 43 |
|  | 35–44 | | | | | | 62 | | | 21 | | 34 | 32 | 13 | 43 | 99 | 19 | 115 | 30 | 116 | 31 |
|  | ≥45 | | | | | | 6 | | | 2 | | 5 | 5 | 5 | 17 | 19 | 4 | 11 | 3 | 19 | 5 |
| **Calendar year of survey** | | | | | |  | | | |  | |  |  |  |  |  |  |  |  |  |  |
|  | 2000–2006 | | | | | | - | | | - | | - | - | - | - | 143 | 27 | 60 | 16 | 37 | 10 |
|  | 2007–2012 | | | | | | 139 | | | 47 | | 45 | 43 | 11 | 37 | 114 | 21 | 94 | 25 | 105 | 28 |
|  | 2013–2017 | | | | | | 155 | | | 53 | | 60 | 57 | 19 | 63 | 271 | 51 | 230 | 60 | 233 | 62 |
| **Ethnicity** | |  | | | | |  | | |  | |  |  |  |  |  |  |  |  |  |  |
|  | Non-Hispanic White | | | | | | 285 | | | 97 | | 103 | 98 | 27 | 90 | 431 | 81 | 298 | 78 | 284 | 76 |
|  | Non-Hispanic Black | | | | | | - | | | - | | - | - | - | - | 12 | 2 | 16 | 4 | 26 | 7 |
|  | Hispanic | | | | | | 7 | | | <1 | | 1 | <1 | 1 | 3 | 61 | 11 | 51 | 13 | 53 | 14 |
|  | Asian or Pacific Islander | | | | | | - | | | - | | 1 | 1 | - | - | 13 | 2 | 7 | 2 | 6 | 2 |
|  | Other | | | | | | - | | | - | | - | - | - | - | 10 | 2 | 12 | 3 | 5 | 1 |
|  | *Missing* | | | | | | *2* | | | *1* | | *-* | *-* | *2* | *7* | *1* | *0* | *0* | *-* | *1* | *0* |
| **Living situation** | | | | | | |  | | |  | |  |  |  |  |  |  |  |  |  |  |
|  | Alone | | | | | | 57 | | | 19 | | 16 | 15 | 8 | 27 | 33 | 6 | 30 | 8 | 32 | 9 |
|  | Other | | | | | | 236 | | | 80 | | 89 | 85 | 22 | 73 | 255 | 48 | 211 | 55 | 230 | 62 |
|  | *Missing* | | | | | | *1* | | | *<1* | | *-* | *-* | *-* | *-* | *240* | *45* | *143* | *37* | *113* | *30* |
| **Education level (highest degree)** ^b^ | | | | | | | | | | |  |  |  |  |  |  |  |  |  |  |  |
|  | Lower | | | | | | 221 | | | 75 | | 90 | 86 | 27 | 90 | 304 | 57 | 221 | 58 | 231 | 62 |
|  | College | | | | | | 73 | | | 25 | | 15 | 14 | 3 | 10 | 220 | 41 | 155 | 41 | 139 | 37 |
|  | *Missing* | | | | | | *-* | | | *-* | | *-* | *-* | *-* | *-* | *4* | *1* | *8* | *2* | *5* | *1* |
| **Household income** ^c^ | | | | | | | |  | |  | |  |  |  |  |  |  |  |  |  |  |
|  | Low | | | | | | 72 | | | 24 | | 21 | 20 | 9 | 30 | 138 | 26 | 99 | 26 | 134 | 36 |
|  | Middle | | | | | | 118 | | | 40 | | 52 | 50 | 12 | 40 | 190 | 36 | 122 | 32 | 123 | 33 |
|  | High | | | | | | 43 | | | 15 | | 10 | 10 | 4 | 13 | 103 | 19 | 62 | 16 | 45 | 12 |
|  | *Missing* | | | | | | *61* | | | *<1* | | *22* | *21* | *5* | *17* | *97* | *18* | *101* | *26* | *73* | *20* |
| **Smoking status** | | |  | | | |  | | |  | |  |  |  |  |  |  |  |  |  |  |
|  | Never | | | | | | 173 | | | 59 | | 61 | 58 | 16 | 53 | 357 | 67 | 251 | 66 | 252 | 68 |
|  | Former | | | | | | 47 | | | 16 | | 22 | 21 | 3 | 10 | 71 | 13 | 82 | 21 | 65 | 17 |
|  | Current | | | | | | 71 | | | 24 | | 22 | 21 | 11 | 37 | 85 | 16 | 45 | 12 | 49 | 13 |
|  | *Missing* | | | | | | *3* | | | *2* | | *-* | *-* | *-* | *-* | *15* | *7* | *6* | *3* | *9* | *5* |
| **Alcohol consumption** ^d^ | | | | | | | | |  |  | |  |  |  |  |  |  |  |  |  |  |
|  | Never/rarely | | | | | | 136 | | | 46 | | 53 | 50 | 18 | 60 | 292 | 55 | 212 | 55 | 247 | 66 |
|  | Weekly | | | | | | 144 | | | 49 | | 45 | 43 | 11 | 37 | 86 | 16 | 62 | 16 | 48 | 13 |
|  | Daily | | | | | | 6 | | | 2 | | 4 | 4 | - | - | 3 | 1 | 0 | - | 3 | 1 |
|  | Frequently | | | | | | 3 | | | 1 | | 1 | 1 | 1 | 3 | 10 | 2 | 6 | 2 | 3 | 1 |
|  | *Missing* | | | | | | *5* | | | *2* | | *2* | *2* | *-* | *-* | *137* | *26* | *104* | *27* | *74* | *20* |
| **Physical activity** ^e^ | | | |  | | |  | | |  | |  |  |  |  |  |  |  |  |  |  |
|  | Inactive | | | | | | 59 | | | 20 | | 22 | 21 | 3 | 10 | 87 | 16 | 83 | 22 | 106 | 28 |
|  | Active | | | | | | 224 | | | 76 | | 77 | 73 | 26 | 87 | 142 | 27 | 120 | 31 | 106 | 28 |
|  | *Missing* | | | | | | *11* | | | *4* | | *6* | *6* | *1* | *3* | *299* | *56* | *181* | *47* | *163* | *44* |

*ALL, acute lymphoblastic leukemia; BMI, body mass index; IQR, interquartile range; SD, standard deviation*

^a^ We matched ALL survivors from North America with survivors in Switzerland on a 1:3 ratio based on sex and attained age.

^b^ Highest degree of education level is categorized as: lower than college graduate/post graduate level and college graduate/post graduate level.

^c^ Household income (income per year) is categorized as: *low*: CCSS baseline (1992–2001): <$20,000, Expansion baseline (2002–2017), Follow-up 2 (2001–2005), follow-up 4 (2007–2009), and follow-up 5 (2014–2016): <$40,000, SCCSS: ≤54,000 Swiss francs; *middle*: CCSS: baseline: $20,000-60,000, other questionnaires: $40,000-100,000, SCCSS:54,000-108,000 Swiss francs, and *high*: CCSS baseline: >$60,000, other questionnaires: >$100,000, SCCSS: >108,000 Swiss francs.

^d^ Alcohol consumption is categorized as: never/rarely; weekly, ≥1 standard drink/week; daily, 1 standard drink/day; frequently, >1 standard drink/day.

^e^ *Physically inactive* is defined as fewer than 150 min of activity per week; physically active is defined as 150 min or more of moderate or 75 min of vigorous physical activity, or a combination of moderate and vigorous intense physical activity per week.

**TABLE S3. Demographic, socio-economic, and lifestyle characteristics of siblings by cohort and BMI category**

|  |  | **BMI** | | | | | | | | | | | | | | | | |
| --- | --- | --- | --- | --- | --- | --- | --- | --- | --- | --- | --- | --- | --- | --- | --- | --- | --- | --- |
|  |  | **Siblings** | | | | | | | | | | | | | | | | |
|  | | **Switzerland** | | | | | | | | | | | **North America**^a^ | | | | | |
|  |  | *n=678* | | | | | | | | | | | *n=2034* | | | | | |
|  |  | **<25 kg/m^2^** | | | | **25-29.9 kg/m^2^** | | | **≥30 kg/m^2^** | | | | **<25 kg/m^2^** | | **25-29.9 kg/m^2^** | | **≥30 kg/m^2^** | |
|  |  | *n=489* | | | | *n=152* | | | *n=37* | | | | *n=966* | | *n=593* | | *n=475* | |
| **Characteristics** | | *n* | | *%* | | *n* | | *%* | *n* | *%* | | | *n* | *%* | *n* | *%* | *n* | *%* |
| **Sex** | |  | |  | |  | |  |  |  | | |  |  |  |  |  |  |
|  | Female | 317 | | 65 | | 59 | | 39 | 26 | 70 | | | 643 | 67 | 282 | 48 | 281 | 59 |
|  | Male | 172 | | 35 | | 93 | | 61 | 11 | 30 | | | 323 | 33 | 311 | 52 | 194 | 41 |
| **Attained age (years)** | | |  |  | |  | |  |  | |  | |  |  |  |  |  |  |
|  | Mean (SD) | 28.3 | | 7.7 | | 32.1 | | 8.0 | 33.8 | 8.2 | | | 29.7 | 7.1 | 32.3 | 7.2 | 33.2 | 7.1 |
|  | Median (IQR) | 26.9 | | (22.3; 32.8) | | 32.0 | | (26.1; 38.4) | 33.8 | (27.4; 37.6) | | | 29.2 | (24.0; 34.9) | 31.7 | (27.0; 36.8) | 32.8 | (27.8; 37.5) |
|  | <25 | 195 | | 40 | | 35 | | 23 | 4 | 11 | | | 286 | 30 | 99 | 17 | 58 | 12 |
|  | 25–34 | 202 | | 41 | | 63 | | 41 | 16 | 43 | | | 442 | 46 | 296 | 50 | 231 | 49 |
|  | 35–44 | 78 | | 16 | | 45 | | 30 | 13 | 35 | | | 218 | 23 | 167 | 28 | 155 | 33 |
|  | ≥45 | 14 | | 3 | | 9 | | 6 | 4 | 11 | | | 20 | 2 | 31 | 5 | 31 | 7 |
| **Calendar year of survey** | | | |  | |  | |  |  | |  | |  |  |  |  |  |  |
|  | 2000–2006 | - | | - | | - | | - | - | - | | | 381 | 39 | 221 | 37 | 143 | 30 |
|  | 2007–2012 | 489 | | 100 | | 152 | | 100 | 37 | 100 | | | 130 | 13 | 93 | 16 | 76 | 16 |
|  | 2013–2017 | - | | - | | - | | - | - | - | | | 455 | 47 | 279 | 47 | 256 | 54 |
| **Ethnicity** | |  | |  | |  | |  |  |  | | |  |  |  |  |  |  |
|  | Non-Hispanic White | 483 | | 99 | | 151 | | 99 | 35 | 95 | | | 833 | 86 | 514 | 87 | 398 | 84 |
|  | Non-Hispanic Black | - | | - | | - | | - | - | - | | | 24 | 2 | 21 | 4 | 23 | 5 |
|  | Hispanic | 3 | | <1 | | 1 | | <1 | - | - | | | 40 | <1 | 25 | 4 | 32 | <1 |
|  | Asian or Pacific Islander | - | | - | | - | | - | 1 | 3 | | | 17 | 2 | 8 | 1 | 2 | <1 |
|  | Other | - | | - | | - | | - | - | - | | | 20 | 2 | 9 | 2 | 8 | 2 |
|  | *Missing* | *3* | | *<1* | | *-* | | *-* | *1* | *3* | | | *32* | *3* | *16* | *3* | *12* | *3* |
| **Living situation** | |  | |  | |  | |  |  |  | | |  |  |  |  |  |  |
|  | Alone | 65 | | 13 | | 24 | | 16 | 8 | 22 | | | 59 | 6 | 30 | 5 | 38 | 8 |
|  | Other | 421 | | 86 | | 125 | | 82 | 29 | 78 | | | 470 | 49 | 343 | 58 | 285 | 60 |
|  | *Missing* | *3* | | *1* | | *3* | | *2* | *-* | *-* | | | *437* | *45* | *220* | *37* | *152* | *32* |
| **Education level (highest degree)**^b^ | | | | | | |  |  | | | |  |  |  |  |  |  |  |
|  | Lower | 212 | | 43 | | 85 | | 56 | 19 | 51 | | | 480 | 50 | 306 | 52 | 278 | 59 |
|  | College | 273 | | 56 | | 66 | | 43 | 15 | 41 | | | 448 | 46 | 264 | 45 | 180 | 38 |
|  | *Missing* | *4* | | *1* | | *1* | | *1* | *3* | *8* | | | *38* | *4* | *23* | *4* | *17* | *4* |
| **Household income**^c^ | | |  | |  | |  |  | | | |  |  |  |  |  |  |  |
|  | Low | 70 | | 14 | | 14 | | 9 | 1 | 3 | | | 194 | 20 | 123 | 21 | 124 | 26 |
|  | Middle | 174 | | 36 | | 68 | | 45 | 17 | 46 | | | 345 | 36 | 250 | 42 | 219 | 46 |
|  | High | 119 | | 24 | | 38 | | 25 | 4 | 11 | | | 269 | 28 | 155 | 26 | 83 | 17 |
|  | *Missing* | *126* | | *26* | | *32* | | *21* | *15* | *41* | | | *158* | *16* | *65* | *11* | *49* | *10* |
| **Smoking status** | |  | |  | |  | |  |  |  | | |  |  |  |  |  |  |
|  | Never | 307 | | 63 | | 88 | | 58 | 21 | 57 | | | 569 | 59 | 336 | 57 | 267 | 56 |
|  | Former | 86 | | 18 | | 31 | | 20 | 8 | 22 | | | 179 | 19 | 130 | 22 | 118 | 25 |
|  | Current | 90 | | 18 | | 33 | | 22 | 7 | 19 | | | 205 | 21 | 118 | 20 | 85 | 18 |
|  | *Missing* | *6* | | *3* | | *-* | | *-* | *1* | *9* | | | *13* | *4* | *9* | *3* | *5* | *3* |
| **Alcohol consumption**^d^ | |  | |  | | |  |  |  |  | | |  |  |  |  |  |  |
|  | Never/rarely | 260 | | 53 | | 70 | | 46 | 27 | 73 | | | 515 | 53 | 311 | 52 | 281 | 59 |
|  | Weekly | 202 | | 41 | | 69 | | 45 | 10 | 27 | | | 192 | 20 | 119 | 20 | 85 | 18 |
|  | Daily | 14 | | 3 | | 6 | | 4 | - | - | | | 18 | 2 | 6 | 1 | 7 | 1 |
|  | Frequently | 11 | | 2 | | 7 | | 5 | - | - | | | 51 | 5 | 32 | 5 | 21 | 4 |
|  | *Missing* | *2* | | *<1* | | *-* | | *-* | *-* | *-* | | | *190* | *20* | *125* | *21* | *81* | *17* |
| **Physical activity**^e^ | |  | |  | |  | |  |  |  | | |  |  |  |  |  |  |
|  | Inactive | 72 | | 15 | | 31 | | 20 | 5 | 14 | | | 121 | 13 | 98 | 17 | 138 | 29 |
|  | Active | 411 | | 84 | | 119 | | 78 | 30 | 81 | | | 307 | 32 | 219 | 37 | 142 | 30 |
|  | *Missing* | *6* | | *1* | | *2* | | *1* | *2* | *5* | | | *538* | *56* | *276* | *47* | *195* | *41* |

*ALL, acute lymphoblastic leukemia; BMI, body mass index; IQR, interquartile range; SD, standard deviation*

^a^ We matched ALL siblings in North America to siblings in Switzerland on a 1:3 ratio based on sex and attained age.

^b^ Highest degree of education level is categorized as: lower than college graduate/post graduate level and college graduate/post graduate level.

^c^ Household income (income per year) is categorized as: *low:* CCSS baseline (1992–2001): <$20,000, Expansion baseline (2002–2017), Follow-up 2 (2001–2005), follow-up 4 (2007–2009), and follow-up 5 (2014–2016): <$40,000, SCCSS: ≤54,000 Swiss francs; *middle*: CCSS: baseline: $20,000-60,000, other questionnaires: $40,000-100,000, SCCSS:54,000-108,000 Swiss francs, and *high*: CCSS baseline: >$60,000, other questionnaires: >$100,000, SCCSS: >108,000 Swiss francs.

^d^ Alcohol consumption is categorized as: never/rarely; weekly, ≥1 standard drink/week; daily, 1 standard drink/day; frequently, >1 standard drink/day.

^e^ *Physically inactive* is defined as fewer than 150 min of activity per week; *physically active* is defined as 150 min or more of moderate or 75 min of vigorous physical activity, or a combination of moderate and vigorous intense physical activity per week.

| **TABLE S4. Clinical characteristics of ALL survivors by cohort and BMI category** | | | | | | | | | | | | | |
| --- | --- | --- | --- | --- | --- | --- | --- | --- | --- | --- | --- | --- | --- |
|  |  |  |  |  |  |  |  |  |  |  |  |  |  |
|  |  | **ALL survivors** | | | | | | | | | | | |
|  | | **Switzerland** | | | | | | **North America**^a^ | | | | | |
|  |  | n=429 | | | | | | *n=1287* | | | | | |
|  |  | **<25 kg/m^2^** | | **25-29.9 kg/m^2^** | | **≥30 kg/m^2^** | | **<25 kg/m^2^** | | **25-29.9 kg/m^2^** | | **≥30 kg/m^2^** | |
|  |  | *n=294* | | *n=105* | | *n=30* | | *n=528* | | *n=384* | | *n=375* | |
| **Characteristics** | | *n* | *%* | *n* | *%* | *n* | *%* | *n* | *%* | *n* | *%* | *n* | *%* |
| **Age at diagnosis (years)** | |  |  |  |  |  |  |  |  |  |  |  |  |
|  | Mean (SD) | 6.2 | 3.9 | 6.4 | 4.3 | 5.8 | 3.6 | 7.4 | 4.7 | 7.9 | 4.9 | 7.3 | 4.8 |
|  | Median (IQR) | 5.1 | (3.1; 8.4) | 4.9 | (3.0; 9.5) | 4.4 | (3.0; 8.1) | 6.0 | (3.5; 10.9) | 6.6 | (3.8; 11.6) | 5.5 | (3.4; 10.9) |
|  | <5 | 144 | 49 | 54 | 51 | 16 | 53 | 222 | 42 | 141 | 37 | 165 | 44 |
|  | 5–9 | 94 | 32 | 27 | 26 | 11 | 37 | 153 | 29 | 116 | 30 | 99 | 26 |
|  | ≥10 | 56 | 19 | 24 | 23 | 3 | 10 | 153 | 29 | 127 | 33 | 111 | 30 |
| **Year of diagnosis** | |  |  |  |  |  |  |  |  |  |  |  |  |
|  | 1976–1980 | 27 | 9 | 18 | 17 | 12 | 40 | 129 | 24 | 87 | 23 | 100 | 27 |
|  | 1981–1985 | 56 | 19 | 22 | 21 | 10 | 33 | 105 | 20 | 66 | 17 | 50 | 13 |
|  | 1986–1990 | 79 | 27 | 29 | 28 | 2 | 7 | 91 | 17 | 80 | 21 | 78 | 21 |
|  | 1991–1995 | 84 | 29 | 28 | 27 | 6 | 20 | 126 | 24 | 90 | 23 | 91 | 24 |
|  | 1996–1999 | 48 | 16 | 8 | 8 | - | - | 77 | 15 | 61 | 16 | 56 | 15 |
| **Time since diagnosis** | |  |  |  |  |  |  |  |  |  |  |  |  |
|  | Mean (SD) | 23.2 | 6.8 | 25.6 | 7.0 | 30.1 | 7.9 | 21.9 | 7.0 | 23.6 | 6.4 | 24.8 | 6.9 |
|  | Median (IQR) | 22.3 | (18.1; 28.1) | 25.5 | (20.4; 30.8) | 30.3 | (24.2; 38.6) | 21.3 | (17.1; 26.0) | 23.1 | (19.0; 27.4) | 23.5 | (19.5; 29.3) |
|  | <10 | 3 | 1 | - | - | - | - | 20 | 4 | 2 | 1 | 1 | <1 |
|  | 11–14 | 30 | 10 | 6 | 6 | 1 | 3 | 61 | 12 | 28 | 7 | 19 | 5 |
|  | 15–19 | 76 | 26 | 18 | 17 | 2 | 7 | 140 | 27 | 86 | 22 | 83 | 22 |
|  | ≥20 | 185 | 63 | 81 | 77 | 27 | 90 | 307 | 58 | 268 | 70 | 272 | 73 |
| **Glucocorticoids** | |  |  |  |  |  |  |  |  |  |  |  |  |
|  | Prednisone | 193 | 66 | 76 | 72 | 26 | 87 | 311 | 59 | 224 | 58 | 233 | 62 |
|  | Dexamethasone | 1 | <1 | - | - | - | - | 23 | 4 | 8 | 2 | 12 | 3 |
|  | Both | 87 | 30 | 25 | 24 | 3 | 10 | 126 | 24 | 105 | 27 | 87 | 23 |
|  | None | 13 | 4 | 3 | 3 | - | - | 18 | 3 | 15 | 4 | 13 | 3 |
|  | *Missing* | - | - | *1* | *1* | *1* | 3 | *50* | *9* | *32* | *8* | *30* | *8* |
| **CRT (gray)** | |  |  |  |  |  |  |  |  |  |  |  |  |
|  | No | 234 | 80 | 79 | 75 | 17 | 57 | 277 | 52 | 176 | 46 | 177 | 47 |
|  | <18 | 30 | 10 | 5 | 5 | 2 | 7 | 15 | 3 | 5 | 1 | 6 | 2 |
|  | ≥18 | 30 | 10 | 21 | 20 | 11 | 37 | 176 | 33 | 160 | 42 | 156 | 42 |
|  | *Missing* | - | - | - | - | - | - | 60 | 11 | 43 | 11 | 36 | 10 |
| **Total body radiation** | |  |  |  |  |  |  |  |  |  |  |  |  |
|  | No | 288 | 98 | 104 | 99 | 30 | 100 | 444 | 84 | 336 | 88 | 335 | 89 |
|  | Yes | 6 | 2 | 1 | 1 | - | - | 24 | 5 | 6 | 2 | 4 | 1 |
|  | *Missing* | - | - | - | - | - | - | 60 | *11* | 42 | *11* | 36 | *10* |
| **HSCT** | |  |  |  |  |  |  |  |  |  |  |  |  |
|  | No | 283 | 96 | 100 | 95 | 30 | 100 | 445 | 84 | 339 | 88 | 332 | 89 |
|  | Yes | 11 | 4 | 5 | 5 | - | - | 32 | 6 | 13 | 3 | 9 | 2 |
|  | *Missing* | - | - | - | - | - | - | 51 | *10* | 32 | *8* | 34 | *9* |
| **Relapse** | |  |  |  |  |  |  |  |  |  |  |  |  |
|  | No | 258 | 88 | 87 | 83 | 30 | 100 | 470 | 89 | 344 | 90 | 342 | 91 |
|  | Yes | 36 | 12 | 18 | 17 | - | - | 58 | 11 | 40 | 10 | 33 | 9 |
| **Second malignancies** | |  |  |  |  |  |  |  |  |  |  |  |  |
|  | No | 275 | 94 | 95 | 90 | 30 | 100 | 512 | 97 | 373 | 97 | 367 | 98 |
|  | Yes | 11 | 4 | 4 | 4 | - | - | 16 | 3 | 11 | 3 | 8 | 2 |
|  | *Missing* | *8* | *3* | *6* | *6* | - | - | - | - | - | - | - | - |

*ALL, acute lymphoblastic leukemia; BMI, body mass index; CRT, cranial radiation therapy; HSCT, hematopoietic stem cell transplantation; IQR, interquartile range; SD, standard deviation*

^a^ We matched ALL survivors from North America with survivors in Switzerland on a 1:3 ratio based on sex and attained age.

| **TABLE S5. Predictors for overweight and obesity in ALL survivors retrieved from univariable multinominal logistic regression; referent: normal BMI**^a^ | | | | | | | | | | | | | | | |
| --- | --- | --- | --- | --- | --- | --- | --- | --- | --- | --- | --- | --- | --- | --- | --- |
|  | | | | | | | | |  |  |  |  |  |  |  |
|  | | | **Overweight vs. Normal** | | | | **Obese vs. Normal** | | |  |  |  |  |  |  |
|  | | | OR (95%CI) | | | | OR (95%CI) | | |  |  |  |  |  |  |
|  | | | Unadjusted | | | *p* | Unadjusted | *p* | |  |  |  |  |  |  |
| **Cohort**^b^ | |  | | |  | | |  | | |  |  |  |  |  |
| Switzerland | | | | 1.00 (ref) | | | <0.001 | 1.00 (ref) | <0.001 | |  |  |  |  |  |
| North America | | | | 1.98 (1.52; 2.58) | | |  | 6.77 (4.54; 10.11) |  | |  |  |  |  |  |
| **Sex** | |  | | |  | | |  | | |  | | |  |  |
| Female | | | | 1.00 (ref) | | | <0.001 | 1.00 (ref) | 0.013 | |  |  |  |  |  |
| Male | | | | 1.86 (1.48; 2.34) | | |  | 1.36 (1.07; 1.74) |  | |  |  |  |  |  |
| **Attained age, y** | | | | |  | | |  | | |  | | |  |  |
| <25 | | | | 1.00 (ref) | | | <0.001 | 1.00 (ref) | <0.001 | |  |  |  |  |  |
| 25–34 | | | | 1.51 (1.13; 2.00) | | |  | 1.58 (1.16; 2.16) |  | |  |  |  |  |  |
| 35–44 | | | | 2.20 (1.60; 3.02) | | |  | 2.53 (1.80; 3.56) |  | |  |  |  |  |  |
| ≥45 | | | | 1.52 (0.78; 2.98) | | |  | 3.04 (1.64; 5.64) |  | |  |  |  |  |  |
| **Calendar year of survey** | | | | |  | | |  | | |  | | |  |  |
| 2000–2006 | | | | 0.68 (0.48; 0.95) | | | 0.070 | 0.48 (0.32; 0.71) | 0.001 | |  |  |  |  |  |
| 2007–2012 | | | | 0.86 (0.67; 1.12) | | |  | 0.83 (0.63; 1.09) |  | |  |  |  |  |  |
| 2013–2017 | | | | 1.00 (ref) | | |  | 1.00 (ref) |  | |  |  |  |  |  |
| **Ethnicity** | | | | |  | | |  | | |  | | |  |  |
| Non-Hispanic White | | | | 1.00 (ref) | | | 0.081 | 1.00 (ref) | <0.001 | |  |  |  |  |  |
| Non-Hispanic Black | | | | 2.19 (1.02; 4.67) | | |  | 4.54 (2.26; 9.12) |  | |  |  |  |  |  |
| Hispanic | | | | 1.35 (0.92; 2.00) | | |  | 1.80 (1.22; 2.65) |  | |  |  |  |  |  |
| Asian or Pacific Islander | | | | 1.19 (0.48; 2.99) | | |  | 1.14 (0.42; 3.12) |  | |  |  |  |  |  |
| Other | | | | 1.97 (0.84; 4.60) | | |  | 1.05 (0.36; 3.09) |  | |  |  |  |  |  |
| **Living situation** | |  | | |  | | |  | | |  | | |  |  |
| Alone | | | | 0.98 (0.67; 1.44) | | | 0.911 | 0.99 (0.68; 1.43) | 0.950 | |  |  |  |  |  |
| Other | | | | 1.00 (ref) | | |  | 1.00 (ref) |  | |  |  |  |  |  |
| **Education level (highest degree)**^c^ | | | | | |  | |  | | |  | | |  |  |
| Lower | | | | 1.00 (ref) | | | 0.818 | 1.00 (ref) | 0.853 | |  |  |  |  |  |
| College | | | | 0.97 (0.77; 1.24) | | |  | 0.98 (0.76; 1.26) |  | |  |  |  |  |  |
| **Household income**^d^ | | | | |  | | |  | | |  | | |  |  |
| Low | | | | 1.26 (0.89; 1.77) | | | 0.459 | 2.12 (1.40; 3.23) | <0.001 | |  |  |  |  |  |
| Middle | | | | 1.14 (0.81; 1.61) | | |  | 1.33 (0.88; 2.01) |  | |  |  |  |  |  |
| High | | | | 1.00 (ref) | | |  | 1.00 (ref) |  | |  |  |  |  |  |
| **Smoking status** | | | | |  | | |  | | |  | | |  |  |
| Never | | | | 1.00 (ref) | | | 0.001 | 1.00 (ref) | 0.156 | |  |  |  |  |  |
| Former | | | | 1.56 (1.15; 2.12) | | |  | 1.19 (0.84; 1.67) |  | |  |  |  |  |  |
| Current | | | | 0.76 (0.55; 1.05) | | |  | 0.78 (0.56; 1.09) |  | |  |  |  |  |  |
| **Alcohol**^e^ | |  | | |  | | |  | | |  | | |  |  |
| Never/rarely | | | | 1.00 (ref) | | | 0.252 | 1.00 (ref) | <0.001 | |  |  |  |  |  |
| Weekly | | | | 0.76 (0.57; 1.00) | | |  | 0.43 (0.31; 0.60) |  | | | |  |  |  |
| Daily | | | | 0.70 (0.22; 2.29) | | |  | 0.46 (0.12; 1.73) |  | | | |  |  |  |
| Frequently | | | | 0.87 (0.35; 2.18) | | |  | 0.47 (0.15; 1.49) |  | | | |  |  |  |
| **Physical activity**^f^ | | | | |  | | |  | | |  | | |  |  |
| Inactive | | | | 1.35 (0.97; 1.87) | | | 0.073 | 1.97 (1.43; 2.71) | <0.001 | |  |  |  |  |  |
| Active | | | | 1.00 (ref) | | |  | 1.00 (ref) |  | |  |  |  |  |  |
| **Age at diagnosis (years)** | | | | |  | | |  | | |  | | |  |  |
| <5 | | | | 0.73 (0.56; 0.97) | | | 0.076 | 0.90 (0.67; 1.21) | 0.344 | |  |  |  |  |  |
| 5–9 | | | | 0.77 (0.57; 1.04) | | |  | 0.79 (0.57; 1.09) |  | |  |  |  |  |  |
| ≥10 | | | | 1.00 (ref) | | |  | 1.00 (ref) |  | |  |  |  |  |  |
| **Year of diagnosis** | | | | |  | | |  | | |  | | |  |  |
| 1976–1980 | | | | 1.00 (ref) | | | 0.748 | 1.00 (ref) | 0.026 | |  |  |  |  |  |
| 1981–1985 | | | | 0.86 (0.59; 1.23) | | |  | 0.55 (0.37; 0.81) |  | |  |  |  |  |  |
| 1986–1990 | | | | 1.01 (0.71; 1.44) | | |  | 0.70 (0.48; 1.00) |  | |  |  |  |  |  |
| 1991–1995 | | | | 0.85 (0.61; 1.20) | | |  | 0.66 (0.47; 0.93) |  | |  |  |  |  |  |
| 1996-1999 | | | | 0.85 (0.58; 1.26) | | |  | 0.65 (0.43; 0.97) |  | |  |  |  |  |  |
| **Time since diagnosis** | | | | |  | | |  | | |  | | |  |  |
| <10 | | | | 0.17 (0.04; 0.73) | | | 0.001 | 0.10 (0.01; 0.74) | <0.001 | |  |  |  |  |  |
| 11–14 | | | | 0.58 (0.38; 0.89) | | |  | 0.40 (0.24; 0.66) |  | |  |  |  |  |  |
| 15–19 | | | | 0.71 (0.54; 0.93) | | |  | 0.67 (0.50; 0.90) |  | |  |  |  |  |  |
| ≥20 | | | | 1.00 (ref) | | |  | 1.00 (ref) |  | |  |  |  |  |  |
| **Glucocorticoids** | | | | |  | | |  | | |  | | |  |  |
| Prednisone | | | | 1.00 (ref) | | | 0.533 | 1.00 (ref) | 0.617 | |  |  |  |  |  |
| Dexamethasone | | | | 0.57 (0.26; 1.25) | | |  | 0.96 (0.47; 1.97) |  | |  |  |  |  |  |
| Both | | | | 1.06 (0.81; 1.38) | | |  | 0.83 (0.61; 1.11) |  | |  |  |  |  |  |
| None | | | | 0.99 (0.54; 1.83) | | |  | 0.82 (0.42; 1.60) |  | |  |  |  |  |  |
| **CRT (gray)** | | |  | |  | | |  | | |  | | |  |  |
| No | | | | 1.00 (ref) | | | <0.001 | 1.00 (ref) | <0.001 | |  |  |  |  |  |
| <18 | | | | 1.07 (0.71; 1.61) | | |  | 1.12 (0.72; 1.74) |  | |  |  |  |  |  |
| ≥18 | | | | 1.76 (1.37; 2.26) | | |  | 2.11 (1.62; 2.75) |  | |  |  |  |  |  |
| **Total body radiation** | | | | |  | | |  | | |  | | |  |  |
| No | | | | 1.00 (ref) | | | 0.155 | 1.00 (ref) | 0.027 | |  |  |  |  |  |
| Yes | | | | 0.55 (0.24; 1.26) | | |  | 0.33 (0.12; 0.88) |  | |  |  |  |  |  |
| **HSCT** | | |  | |  | | |  | | |  | | |  |  |
| No | | | | 1.00 (ref) | | | 0.274 | 1.00 (ref) | 0.073 | |  |  |  |  |  |
| Yes | | | | 0.73 (0.42; 1.28) | | |  | 0.51 (0.25; 1.06) |  | |  |  |  |  |  |
| **Relapse** | | |  | |  | | |  |  | |  | | |  |  |
| No | | | | 1.00 (ref) | | | 0.643 | 1.00 (ref) | 0.123 | |  |  |  |  |  |
| Yes | | | | 1.09 (0.76; 1.55) | | |  | 0.72 (0.47; 1.09) |  | |  |  |  |  |  |
| **Second malignancies** | | | | |  | | |  | | |  | | | |  |
| No | | | | 1.00 (ref) | | | 0.722 | 1.00 (ref) | 0.146 | |  |  |  |  |  |
| Yes | | | | 0.89 (0.46; 1.70) | | |  | 0.55 (0.25; 1.23) |  | |  |  |  |  |  |

*ALL, acute lymphoblastic leukemia; BMI, body mass index; CRT, cranial radiation therapy; HSCT, hematopoietic stem cell transplantation; OR, odds ratio; ref, reference*

^a^ We excluded survivors who had underweight (SCCSS: n=29, CCSS: n=39)

^b^ We matched ALL survivors from North America with survivors in Switzerland on a 1:3 ratio based on sex and attained age.

^c^ Highest degree of education level is categorized as: lower than college graduate/post graduate level and college graduate/post graduate level.

^d^ Household income (income per year) is categorized as: *low*: CCSS baseline (1992–2001): <$20,000, Expansion baseline (2002–2017), Follow-up 2 (2001–2005), follow-up 4 (2007–2009), and follow-up 5 (2014–2016): <$40,000, SCCSS: ≤54,000 Swiss francs; *middle*: CCSS: baseline: $20,000-60,000, other questionnaires: $40,000-100,000, SCCSS:54,000-108,000 Swiss francs, and *hig*h: CCSS baseline: >$60,000, other questionnaires: >$100,000, SCCSS: >108,000 Swiss francs

^e^ Alcohol consumption is categorized as: never/rarely; weekly, ≥1 standard drink/week; daily, 1 standard drink/day; frequently, >1 standard drink/day.

^f^ *Physically inactive* is defined as fewer than 150 min of activity per week; *Physically active* is defined as 150 min or more of moderate or 75 min of vigorous physical activity, or a combination of moderate and vigorous intense physical activity per week.

**TABLE S6. Predictors for overweight and obesity in ALL survivors comparing the CCSS (North America) with the SCCSS (Switzerland) retrieved from multivariable multinominal logistic regression**^a.b^**; referent: normal BMI**

|  |  | **SCCSS (Switzerland)^a^** | | | | | **CCSS (North America)**^a,c^ | | | |
| --- | --- | --- | --- | --- | --- | --- | --- | --- | --- | --- |
|  |  | *n=400* | | | | | *n=1249* | | | |
|  |  | **Overweight vs. Normal** | | **Obese vs. Normal** | | **Overweight vs. Normal** | | | **Obese vs. Normal** | |
| **Characteristics** | | OR (95%CI) | *p* | OR (95%CI) | *p* | OR (95%CI) | | *p* | OR (95%CI) | *p* |
| **Sex** | |  |  |  |  |  | |  |  |  |
|  | Female | 1.00 (ref) | <0.001 | 1.00 (ref) | 0.072 | 1.00 (ref) | | <0.001 | 1.00 (ref) | 0.002 |
|  | Male | 3.59 (2.08; 6.21) |  | 2.38 (0.92; 6.14) |  | 1.78 (1.34; 2.38) | |  | 1.58 (1.18; 2.12) |  |
| **Attained age (years)** | |  |  |  |  |  | |  |  |  |
|  | <25 | 1.00 (ref) | 0.415 | 1.00 (ref) | 0.001 | 1.00 (ref) | | 0.004 | 1.00 (ref) | <0.001 |
|  | 25–34 | 1.27 (0.67; 2.41) |  | 1.46 (0.39; 5.37) |  | 1.58 (1.10; 2.27) | |  | 2.07 (1.41; 3.05) |  |
|  | 35–44 | 1.78 (0.86; 3.66) |  | 5.45 (1.41; 21.11) |  | 2.09 (1.34; 3.26) | |  | 3.28 (2.06; 5.22) |  |
|  | ≥45 | 2.24 (0.48; 10.37) |  | 41.39 (5.14; 333.11) |  | 0.96 (0.40; 2.31) | |  | 3.26 (1.42; 7.45) |  |
| **Ethnicity** | |  |  |  |  |  | |  |  |  |
|  | Non-Hispanic White | 1.00 (ref) | 0.861 | 1.00 (ref) | 0.433 | 1.00 (ref) | | 0.281 | 1.00 (ref) | 0.009 |
|  | Non-Hispanic Black | - |  | - |  | 1.92 (0.87; 4.23) | |  | 3.24 (1.55; 6.77) |  |
|  | Hispanic | 0.53 (0.06; 5.13) |  | 5.31 (0.42; 66.72) |  | 1.37 (0.89; 2.11) | |  | 1.56 (1.01; 2.41) |  |
|  | Asian or Pacific Islander | - |  | - |  | 1.00 (0.37; 2.71) | |  | 0.86 (0.29; 2.53) |  |
|  | Other | - |  | - |  | 1.58 (0.64; 3.92) | |  | 0.68 (0.22; 2.14) |  |
| **Household income**^d^ | |  |  |  |  |  | |  |  |  |
|  | Low | 1.64 (0.66; 4.04) | 0.186 | 0.83 (0.16; 4.24) | 0.973 | 1.44 (0.94; 2.23) | | 0.179 | 2.36 (1.46; 3.81) | <0.001 |
|  | Middle | 2.10 (0.91; 4.86) |  | 0.86 (0.19; 3.86) |  | 1.07 (0.72; 1.59) | |  | 1.42 (0.91; 2.22) |  |
|  | High | 1.00 (ref) |  | 1.00 (ref) |  | 1.00 (ref) | |  | 1.00 (ref) |  |
| **Smoking status** | |  |  |  |  |  | |  |  |  |
|  | Never | 1.00 (ref) | 0.239 | 1.00 (ref) | 0.158 | 1.00 (ref) | | 0.010 | 1.00 (ref) | 0.318 |
|  | Former | 1.66 (0.86; 3.23) |  | 1.14 (0.29; 4.54) |  | 1.56 (1.07; 2.27) | |  | 1.22 (0.82; 1.83) |  |
|  | Current | 0.88 (0.47; 1.65) |  | 2.64 (0.96; 7.21) |  | 0.72 (0.46; 1.11) | |  | 0.81 (0.52; 1.26) |  |
| **Alcohol consumption**^e^ | |  |  |  |  |  | |  |  |  |
|  | Never/rarely | 1.00 (ref) | 0.147 | 1.00 (ref) | 0.406 | 1.00 (ref) | | 0.998 | 1.00 (ref) | 0.445 |
|  | Weekly | 0.54 (0.31; 0.95) |  | 0.43 (0.16; 1.16) |  | 0.99 (0.63; 1.56) | |  | 0.73 (0.47; 1.15) |  |
|  | Daily | 0.82 (0.18; 3.77) |  | - |  | - | |  | 1.03 (0.17; 6.18) |  |
|  | Frequently | 0.25 (0.02; 2.68) |  | 0.50 (0.03; 9.51) |  | 0.90 (0.31; 2.64) | |  | 0.50 (0.11; 2.16) |  |
| **Physical activity**^f^ | |  |  |  |  |  | |  |  |  |
|  | Inactive | 1.25 (0.67; 2.32) | 0.477 | 0.43 (0.11; 1.71) | 0.232 | 1.22 (0.78; 1.92) | | 0.376 | 1.52 (1.06; 2.17) | 0.022 |
|  | Active | 1.00 (ref) |  | 1.00 (ref) |  | 1.00 (ref) | |  | 1.00 (ref) |  |
| **Age at diagnosis (years)** | |  | |  |  |  | |  |  |  |
|  | <5 | 1.02 (0.52; 2.00) | 0.095 | 7.34 (1.50; 36.01) | 0.046 | 0.91 (0.62; 1.32) | | 0.783 | 1.34 (0.91; 1.97) | 0.289 |
|  | 5–9 | 0.55 (0.27; 1.13) |  | 3.99 (0.84; 18.87) |  | 1.02 (0.70; 1.48) | |  | 1.10 (0.74; 1.63) |  |
|  | ≥10 | 1.00 (ref) |  | 1.00 (ref) |  | 1.00 (ref) | |  | 1.00 (ref) |  |
| **CRT (gray)** | |  |  |  |  |  | |  |  |  |
|  | No | 1.00 (ref) | 0.025 | 1.00 (ref) | 0.098 | 1.00 (ref) | | 0.171 | 1.00 (ref) | 0.560 |
|  | <18 | 0.40 (0.14; 1.16) |  | 1.74 (0.33; 9.22) |  | 1.44 (0.80; 2.56) | |  | 1.32 (0.73; 2.37) |  |
|  | ≥18 | 2.04 (1.01; 4.14) |  | 3.10 (1.08; 8.89) |  | 1.32 (0.96; 1.81) | |  | 1.14 (0.82; 1.58) |  |
| **Total body radiation** | |  |  |  |  |  | |  |  |  |
|  | No | 1.00 (ref) | 0.204 | 1.00 (ref) | - | 1.00 (ref) | | 0.056 | 1.00 (ref) | 0.005 |
|  | Yes | 0.24 (0.03; 2.18) |  | - |  | 0.36 (0.13; 1.03) | |  | 0.21 (0.07; 0.62) |  |

*ALL, acute lymphoblastic leukemia; BMI, body mass index; CRT, cranial radiation therapy; HSCT, hematopoietic stem cell transplantation; OR, odds ratio; ref, reference*

^a^ We excluded survivors that had underweight (n=29 SCCSS, N=39 CCSS)

^b^ Adjusted for all variables listed

^c^ We matched ALL survivors from North America with survivors in Switzerland on a 1:3 ratio based on sex and attained age.

^d^ Household income (income per year) is categorized as: *low*: CCSS baseline (1992–2001): <$20,000, Expansion baseline (2002–2017), Follow-up 2 (2001–2005), follow-up 4 (2007–2009), and follow-up 5 (2014–2016): <$40,000, SCCSS: ≤54,000 Swiss francs; *middle*: CCSS: baseline: $20,000-60,000, other questionnaires: $40,000-100,000, SCCSS:54,000-108,000 Swiss francs, and *high*: CCSS baseline: >$60,000, other questionnaires: >$100,000, SCCSS: >108,000 Swiss francs.

^e^ Alcohol consumption is categorized as: never/rarely; weekly, ≥1 standard drink/week; daily, 1 standard drink/day; frequently, >1 standard drink/day.

^f^ *Physically inactive* is defined as fewer than 150 min of activity per week; *physically active* is defined as 150 min or more of moderate or 75 min of vigorous physical activity, or a combination of moderate and vigorous intense physical activity per week.

**TABLE S7. Predictors for overweight and obesity in ALL survivors (retrieved from multivariable linear regression)**^a^

|  |  | **BMI (kg/m^2^)** | |  |  |  |
| --- | --- | --- | --- | --- | --- | --- |
|  |  | β (95%CI) | *p*^b^ |  |  |  |
| **Cohort** | |  |  |  |  |  |
|  | Switzerland | Ref | <0.001 |  |  |  |
|  | North America^c^ | 3.30 (2.58; 4.03) |  |  |  |  |
| **Sex** | |  |  |  |  |  |
|  | Female | Ref | <0.001 |  |  |  |
|  | Male | 1.10 (0.53; 1.66) |  |  |  |  |
| **Attained age, y** | |  |  |  |  |  |
|  | <25 | Ref | <0.001 |  |  |  |
|  | 25–34 | 1.67 (0.96; 2.38) |  |  |  |  |
|  | 35–44 | 2.96 (2.12; 3.80) |  |  |  |  |
|  | ≥45 | 4.04 (2.41; 5.67) |  |  |  |  |
| **Ethnicity** | |  |  |  |  |  |
|  | Non-Hispanic White | Ref | <0.001 |  |  |  |
|  | Non-Hispanic Black | 2.96 (1.38; 4.55) |  |  |  |  |
|  | Hispanic | 1.16 (0.22; 2.10) |  |  |  |  |
|  | Asian or Pacific Islander | 0.43 (-1.79; 2.65) |  |  |  |  |
|  | Other | -0.98 (-3.22; 1.25) |  |  |  |  |
| **Household income**^d^ | |  |  |  |  |  |
|  | Low | 1.79 (0.82; 2.76) | <0.001 |  |  |  |
|  | Middle | 0.84 (-0.04; 1.73) |  |  |  |  |
|  | High | Ref |  |  |  |  |
| **Smoking status** | | | |  |  |  |
|  | Never | Ref | 0.828 |  |  |  |
|  | Former | 0.09 (-0.67; 0.84) |  |  |  |  |
|  | Current | -0.20 (-0.99; 0.58) |  |  |  |  |
| **Alcohol**^e^ | |  |  |  |  |  |
|  | Never/rarely | Ref | 0.133 |  |  |  |
|  | Weekly | -0.83 (-1.54; -0.12) |  |  |  |  |
|  | Daily | 0.56 (-2.38; 3.49) |  |  |  |  |
|  | Frequently | -0.54 (-2.87; 1.79) |  |  |  |  |
| **Physical activity**^f^ | |  |  |  |  |  |
|  | Inactive | 0.53 (-0.23; 1.29) | 0.168 |  |  |  |
|  | Active | Ref |  |  |  |  |
| **Age at diagnosis (years)** | | |  |  |  |  |
|  | <5 | 0.88 (0.14; 1.62) | 0.065 |  |  |  |
|  | 5–9 | 0.51 (-0.24; 1.27) |  |  |  |  |
|  | ≥10 | Ref |  |  |  |  |
| **CRT (gray)** | |  |  |  |  |  |
|  | No | Ref | 0.242 |  |  |  |
|  | <18 | 0.81 (-0.25; 1.87) |  |  |  |  |
|  | ≥18 | 0.36 (-0.29; 1.02) |  |  |  |  |
| **Total body radiation** | |  |  |  |  |  |
|  | No | Ref | <0.001 |  |  |  |
|  | Yes | -3.80 (-5.52; -2.09) |  | | | |

*ALL, acute lymphoblastic leukemia; BMI, body mass index; CRT, cranial radiation therapy; HSCT, hematopoietic stem cell transplantation; OR, odds ratio; ref, reference*

^a^ Adjusted for all variables listed.

^b^ p-value calculated from F-test.

^c^ We matched ALL survivors from North America with survivors in Switzerland on a 1:3 ratio based on sex and attained age.

^d^ Household income (income per year) is categorized as: *low*: CCSS baseline (1992–2001): <$20,000, Expansion baseline (2002–2017), Follow-up 2 (2001–2005), follow-up 4 (2007–2009), and follow-up 5 (2014–2016): <$40,000, SCCSS: ≤54,000 Swiss francs; *middle*: CCSS: baseline: $20,000-60,000, other questionnaires: $40,000-100,000, SCCSS:54,000-108,000 Swiss francs, and *high*: CCSS baseline: >$60,000, other questionnaires: >$100,000, SCCSS: >108,000 Swiss francs

^e^ Alcohol consumption is categorized as: never/rarely; weekly, ≥1 standard drink/week; daily, 1 standard drink/day; frequently, >1 standard drink/day.

^f^ *Physically inactive* is defined as fewer than 150 min of activity per week; *Physically active* is defined as 150 min or more of moderate or 75 min of vigorous physical activity, or a combination of moderate and vigorous intense physical activity per week.

| **TABLE S8. Interaction of cohort with sociodemographic and clinical characteristics (retrieved from multivariable multinominal logistic regression; referent: normal BMI)** | | | | | | |
| --- | --- | --- | --- | --- | --- | --- |
|  | **p values for interactions**^a^ | | | | |  |
|  | | **North American vs Swiss ALL survivors**^b,c^ | | **North American vs Swiss siblings**^c.d^ | |  |
|  | | *n=1647* | | *n=2636* | |  |
| **Characteristics** | | **Overweight vs normal**^e^ | **Obese vs normal**^e^ | **Overweight vs normal**^e^ | **Obese vs normal**^e^ |  |
| **Sociodemographic** | |  |  |  |  |  |
| Sex | | 0.120 | 0.298 | 0.466 | 0.059 |  |
| Attained age (<25, 25–34, 35–44, ≥45 years) | | 0.404 | 0.050 | 0.523 | 0.604 |  |
| Calendar year of survey (<2013, ≥2013) | | 0.112 | 0.418 | - | - |  |
| Ethnicity (Non-Hispanic White, other) | | 0.890 | 0.751 | 0.987 | 0.310 |  |
| Living situation (Alone, Other) | | 0.182 | 0.573 | 0.345 | 0.498 |  |
| Education level (Lower, College)^f^ | | 0.678 | 0.785 | 0.154 | 0.836 |  |
| Household income (Low, Middle, High)^g^ | | 0.173 | 0.657 | 0.228 | 0.487 |  |
| Smoking status (Never, Former, Current) | | 0.889 | 0.178 | 0.728 | 0.856 |  |
| Alcohol consumption (Never/rarely, More)^h^ | | 0.371 | 0.699 | 0.108 | 0.169 |  |
| Physical activity (Inactive, Active)^i^ | | 0.943 | 0.078 | 0.600 | 0.094 |  |
| **Clinical** | |  |  |  |  |  |
| Age at diagnosis, (<5, ≥5 years) | | 0.156 | 0.561 | NA | NA |  |
| Year of diagnosis, (<1980,1981–1985, 1986–1990, 1991–1995, ≥1996) | | 0.152 | 0.040 | NA | NA |  |
| Time since diagnosis, (<20, ≥20 years) | | 0.723 | 0.192 | NA | NA |  |
| Glucocorticoids (Prednisone, Dexamethasone, Both, None) | | 0.556 | 0.351 | NA | NA |  |
| CRT (No, <18 Gy, ≥18 Gy) | | 0.055 | 0.035 |  |  |  |
| Total body radiation (No, Yes) | | 0.647 | - | NA | NA |  |
| HSCT (No, Yes) | | 0.355 | - | NA | NA |  |
| Relapse (No, Yes) | | 0.654 |  | NA | NA |  |
| Second malignancies (No, Yes) | | 0.927 | - | NA | NA |  |

*ALL, acute lymphoblastic leukemia; BMI, body mass index; CRT, cranial radiation therapy; HSCT, hematopoietic stem cell transplantation; NA, not applicable*

^a^ We calculated the p-value for interaction with the Wald test

^b^ Adjusted for sex, attained age, ethnicity, household income, smoking status, alcohol consumption, physical activity, age at diagnosis, CRT, and total body irradiation

^c^ We matched ALL survivors/siblings from North America with survivors/siblings in Switzerland on a 1:3 ratio based on sex and attained age

^d^ Adjusted for sex, attained age, ethnicity, household income, smoking status, alcohol consumption, and physical activity

^e^ We excluded those that had underweight (survivors: n=29 SCCSS, n=39 CCSS; siblings: n=12 SCCSS, n=64 CCSS)

^f^ Highest degree of education level is categorized as: lower than college graduate/post graduate level and college graduate/post graduate level.

^g^ Household income (income per year) is categorized as: low: CCSS baseline (1992–2001): <$20,000, Expansion baseline (2002–2017), Follow-up 2 (2001–2005), follow-up 4 (2007–2009), and follow-up 5 (2014–2016): <$40,000, SCCSS: ≤54,000 Swiss francs; *middle*: CCSS: baseline: $20,000-60,000, other questionnaires: $40,000-100,000, SCCSS:54,000-108,000 Swiss francs, and *high*: CCSS baseline: >$60,000, other questionnaires: >$100,000, SCCSS: >108,000 Swiss francs

^h^ Alcohol consumption is categorized as: never/rarely; weekly, ≥1 standard drink/week; daily, 1 standard drink/day; frequently, >1 standard drink/day

^i^ *Physically inactive* is defined as fewer than 150 min of activity per week; *physically active* is defined as 150 min or more of moderate or 75 min of vigorous physical activity, or a combination of moderate and vigorous intense physical activity per week

**SUPPLEMENTAL METHODS**

**CCSS Institutions:**

1. St. Jude Children's Research Hospital, Memphis, TN
2. Ann & Robert H. Lurie Children’s Hospital of Chicago, Chicago, IL
3. Children's Healthcare of Atlanta/Emory University, Atlanta, GA
4. Children's Hospitals and Clinics of Minnesota, Minneapolis, MN
5. Children's Hospital of Colorado, Aurora CO
6. Children's Hospital of Los Angeles, Los Angeles, CA
7. Children's Hospital of Orange County, Orange, CA
8. Children's Hospital of Philadelphia, Philadelphia, PA
9. Children's Hospital of Pittsburgh, Pittsburgh, PA
10. Children's National Medical Center, Washington, DC
11. Cincinnati Children's Hospital Medical Center, Cincinnati, OH
12. City of Hope Medical Center, Los Angeles, CA
13. Cook Children's Medical Center, Ft. Worth, TX
14. Dana Farber Cancer Institute/Children's Hospital, Boston, MA
15. Fred Hutchinson Cancer Research Center, Seattle, WA
16. Hospital for Sick Children, Toronto, ON
17. Mayo Clinic, Rochester, MN
18. Memorial Sloan Kettering Cancer Center, New York, NY
19. Miller Children's Hospital, Long Beach, CA
20. National Cancer Institute, Bethesda, MD
21. Nationwide Children's Hospital, Columbus, OH
22. Riley Hospital for Children, Indianapolis, IN
23. Roswell Park Cancer Institute, Buffalo, NY
24. St. Louis Children's Hospital, St. Louis, MO
25. Seattle Children's Hospital, Seattle, WA
26. Stanford University School of Medicine, Palo Alto, CA
27. Texas Children's Hospital, Houston, TX
28. University of Alabama, Birmingham, AL
29. University of California-Los Angeles, Los Angeles, CA
30. University of California-San Francisco, San Francisco, CA
31. University of Chicago, Chicago, IL

**Swiss Childhood Cancer Survivor Study institutions:**

1. University Children's Hospital Basel, Basel
2. University Children's Hospital Bern, Bern
3. University Children's Hospital Geneva, Geneva
4. University Children's Hospital Lausanne, Lausanne
5. University Children's Hospital Zurich, Zurich
6. Cantonal Children’s Hospital Aarau, Aarau
7. Cantonal Children’s Hospital Bellinzona, Bellinzona
8. Cantonal Children’s Hospital Lucerne, Lucerne
9. Cantonal Children’s Hospital St. Gallen, St. Gallen
